# A methyltransferase‐like 14/miR‐99a‐5p/tribble 2 positive feedback circuit promotes cancer stem cell persistence and radioresistance via histone deacetylase 2‐mediated epigenetic modulation in esophageal squamous cell carcinoma

**DOI:** 10.1002/ctm2.545

**Published:** 2021-09-15

**Authors:** Zhenchuan Liu, Kaiqing Wu, Shaorui Gu, Wenli Wang, Shiliang Xie, Tiancheng Lu, Lei Li, Chenglai Dong, Xishi Wang, Yongxin Zhou

**Affiliations:** ^1^ Department of Thoracic Surgery, Shanghai Tongji Hospital, School of Medicine Tongji University Shanghai P.R. China

**Keywords:** cancer stem‐like cells, esophageal squamous cell carcinoma, positive feedback loop, radioresistance

## Abstract

**Background:**

Esophageal squamous cell carcinoma (ESCC) is a highly aggressive and treatment‐resistant tumor. The biological implications and molecular mechanism of cancer stem‐like cells (CSCs) in ESCC, which contribute to therapeutic resistance such as radioresistance, remain elusive.

**Methods:**

Quantitative real‐time polymerase chain reaction, western blotting, immunohistochemistry, and in situ hybridization assays were used to detect methyltransferase‐like 14 miR‐99a‐5p tribble 2 (METTL14/miR‐99a‐5p/TRIB2) expression in ESCC. The biological functions of METTL14/miR‐99a‐5p/TRIB2 were demonstrated in vitro and in vivo. Mass spectrum analysis was used to identify the downstream proteins regulated by TRIB2. Chromatin immunoprecipitation (IP), IP, N^6^‐methyladenosine (m^6^A)‐RNA IP, luciferase reporter, and ubiquitination assays were employed to explore the molecular mechanisms underlying this feedback circuit and its downstream pathways.

**Results:**

We found that miR‐99a‐5p was significantly decreased in ESCC. miR‐99a‐5p inhibited CSCs persistence and the radioresistance of ESCC cells, and miR‐99a‐5p downregulation predicted an unfavorable prognosis of ESCC patients. Mechanically, we unveiled a METTL14‐miR‐99a‐5p‐TRIB2 positive feedback loop that enhances CSC properties and radioresistance of ESCC cells. METTL14, an m^6^A RNA methyltransferase downregulated in ESCC, suppresses TRIB2 expression via miR‐99a‐5p‐mediated degradation of TRIB2 mRNA by targeting its 3′ untranslated region, whereas TRIB2 induces ubiquitin‐mediated proteasomal degradation of METTL14 in a COP1‐dependent manner. METTL14 upregulates miR‐99a‐5p by modulating m^6^A‐mediated, DiGeorge critical region 8‐dependent pri‐mir‐99a processing. Hyperactivation of TRIB2 resulting from this positive circuit was closely correlated with radioresistance and CSC characteristics. Furthermore, TRIB2 activates HDAC2 and subsequently induces p21 epigenetic repression through Akt/mTOR/S6K1 signaling pathway activation. Pharmacologic inhibition of HDAC2 effectively attenuates the TRIB2‐mediated effect both in vitro and in patient‐derived xenograft models.

**Conclusion:**

Our data highlight the presence of the METTL14/miR‐99a‐5p/TRIB2 axis and show that it is positively associated with CSC characteristics and radioresistance of ESCC, suggesting potential therapeutic targets for ESCC treatment.

## Graphical abstract

1. METTL14 upregulates miR‐99a‐5p by promoting m^6^A‐mediated, DGCR8‐dependent

2. miR‐99a‐5p induces degradation of TRIB2 mRNA by targeting its 3′ UTR.

3. TRIB2 induces ubiquitin‐mediated proteasomal degradation of METTL14 in a COP1‐dependent manner.

4. METTL14/miR‐99a‐5p/TRIB2 constitutes a positive feedback loop that enhances CSC properties and radioresistance of ESCC cells through inducing HDAC2‐mediated p21 epigenetic repression dependent on Akt/mTOR/S6K1 pathway.

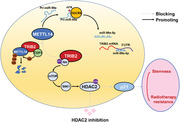

**Abbreviations**
3′ UTR: 3′, untranslated region
Act‐D: actinomycin D
ChIP: chromatin immunoprecipitation
CHX: cycloheximide
CSC: cancer stem‐like cell
DICER: DiGeorge critical region 8‐dependent
ESCC: esophageal squamous cell carcinoma
GEO: Gene Expression Omnibus
HDAC: histone deacetylase
IHC: immunohistochemistry
ISH: in situ hybridization
MAPK: mitogen‐activated protein kinase
MeRIP: m^6^A‐RNA immunoprecipitation
METTL14: methyltransferase‐like 14
METTL3: methyltransferase‐like 3
miRNAs: microRNAs
mTOR: mechanistic target of rapamycin kinase
Mut: mutated type
OS: overall survival
PANTs: paired adjacent nontumorous tissues
PDX: patient‐derived xenograft
PFS: progression‐free survival
RT‐PCR: real‐time polymerase chain reaction
S6K1: ribosomal protein S6 kinase B1
SCA: santacruzamate A
SDS: sodium dodecyl sulfate
SI: staging index
TCGA: The Cancer Genome Atlas
WT: wild type

## 1 INTRODUCTION

Esophageal squamous cell carcinoma (ESCC) accounts for the majority of esophageal cancers and exhibits a higher incidence in developing nations.^1^ A comprehensive approach consisting of neoadjuvant chemoradiation followed by radical surgical resection is generally used to treat locally advanced ESCC. Unfortunately, most patients die from metastasis and recurrence due to inadequate treatment response.^2^

The cancer stem‐like cell (CSC) paradigm suggests that a subset of cancer cells in solid tumors exhibit the characteristics of normal stem cells and thus have the capacity for self‐renewal and differentiation.^3^ CSCs are believed to possess high carcinogenic and metastatic potential and to be resistant to conventional antitumor treatment in many malignancies, including ESCC.^4^ CSCs have also been identified as being responsible for the radioresistance of ESCC.^5^ Therefore, discovering the complex molecular mechanisms that produce the CSC state would be especially helpful in identifying potentially effective strategies for overcoming radioresistance in ESCC.

MicroRNAs (miRNAs) are small noncoding RNAs that act as regulators of gene expression through the inhibition of translation and/or as negative modulators of mRNA stability by binding to the 3′ untranslated region (3′ UTR) of target mRNAs.^6^ Notably, the dysregulation of miRNAs plays an important role in carcinogenesis or metastasis in ESCC^7^ as well as in other types of cancers.^8^ To date, miRNAs involved in regulating the CSC properties of ESCC have been rarely characterized.

This study aimed to identify miRNAs involved in the regulation of cancer cell stemness persistence by analyzing differentially expressed miRNAs between CSCs and non‐CSCs of ESCC cells. Among these, we found that miR‐99a‐5p (mircoRNA‐99a‐5p) exhibited the most prominent effect on suppressing the CSC properties of ESCC through promoting tribble 2 (TRIB2) degradation. Tribbles (TRIB) family are a set of serine/threonine kinase‐like proteins that act as scaffolding proteins and signaling mediators in several signaling pathways.^9^ Recently, TRIB3 has been reported to confer radiotherapy resistance by stabilizing Tafazzin (TAZ),^10^ suggesting an important pro‐oncogenic role of the tribble family in ESCC. We report here that decreased miR‐99a‐5p and overexpression of its downstream target TRIB2 played essential roles in establishing CSC properties and radioresistance in ESCC through the activation of histone deacetylase 2 (HDAC2). Furthermore, METTL14 positively regulated pri‐mir‐99a processing in an methyladenosine (m^6^A) methylation‐induced, DiGeorge critical region 8 (DGCR8)‐dependent manner. Methyltransferase‐like 14 (METTL14) activity was suppressed in ESCC due to TRIB2‐assisted, ubiquitin‐mediated proteolysis. We thus postulated the existence of a METTL14/miR‐99a‐5p/TRIB2 feedback circuit and further explored the rationale for this circuit as a therapeutic target for ESCC.

## 2 METHODS AND MATERIALS

### 2.1 Human specimens

Cohort I. For real‐time polymerase chain reaction (RT‐PCR) analysis, a total of 26 fresh ESCC samples and paired adjacent nontumorous tissues were collected from Shanghai Tongji Hospital.

Cohort II. For immunohistochemistry (IHC) and in situ hybridization (ISH) analyses, 86 ESCC and corresponding adjacent 3‐μm sections of formalin‐fixed paraffin‐embedded tissues were collected from Shanghai Tongji Hospital. None of the patients in this study received any systemic treatment before the samples were collected.

Cohort III. A total of 78 consecutive ESCC patients who received definite radiotherapy from 2011 to 2013 at Shanghai Tongji Hospital were enrolled in this study.

This study was approved by the Medical Ethics Committee of Shanghai Tongji Hospital. Written informed consent was obtained from each patient prior to inclusion. The clinical characteristics of the patients are listed in Tables S5–S7.

### 2.2 Cell culture

The HEK293T cell line, a human normal esophageal squamous epithelial cell line (Het‐1a) and ESCC cell lines (Eca109, EC9706, Kyse150, Kyse410, and TE‐1) were obtained from the Cell Bank of the Chinese Academy of Sciences (Shanghai, China). All cell lines were authenticated by the short tandem repeat method. Cells were cultured in RPMI 1640 medium (HyClone) supplemented with 10% fetal bovine serum (Gibco) and 1% penicillin and streptomycin (HyClone) at 37°C in an incubator with 5% CO_2_. All cells were checked for mycoplasma contamination.

### 2.3 Antibodies and reagents

Chaetocin (ab144534) and the following antibodies were purchased from Abcam: Homeobox Transcription Factor Nanog (NANOG, ab109250); Octamer‐Binding Protein 4 (OCT4, ab181557); Polycomb Ring Finger (BM1‐1, ab126783); ATP‐Binding Cassette Transporter G2 (ABCG2, ab207732); CD90 (ab133350); CD271 (ab52987); TRIB2 (ab117981); Aldehyde Dehydrogenase 1 Family Member A1 (ALDH1, ab52492); SRY‐Box Transcription Factor 2 (SOX2, ab92494); ATM (Ataxia Telangiectasia Mutated, ab32420); p‐ATM (S1981, ab81292); Methyltransferase 3 (METTL3, ab195352); WT1 Associated Protein (WTAP, ab195380); alkylation repair homolog protein 5 (ab195377); fat‐mass obesity‐associated protein (ab126605); Checkpoint Kinase 2 (CHK2, ab109413); p‐CHK2 (T68, ab32148); caspase‐3 (ab32150); cleaved caspase‐3 (ab32042); Poly(ADP‐Ribose) Polymerase 1 (PARP1, ab74290); cleaved PARP (ab32064); Glyceraldehyde‐3‐Phosphate Dehydrogenase (GAPDH, ab8245); H2A.X Variant Histone (S139) (γ‐H2AX, ab81299); Methyltransferase 14 (METTL14, ab220030); DiGeorge Syndrome Critical Region Gene 8 (DGCR8, ab191875); Myc tag (ab32); DDDDK tag (ab205606); 6X His tag (ab18184); V5 tag (ab206566); ubiquitin (ab134953); Constitutive Photomorphogenesis Protein 1 Homolog (COP1, ab56400); histone H3 (ab1791); histone H4 (ab177840); H3K9Ac (ab177177); H3K14Ac (ab52946); ac‐H4 (ab51997); AKT Serine/Threonine Kinase (Akt, ab8805); p‐Akt (S473, ab81283); Histone Deacetylase 2 (HDAC2, ab32117); p‐HDAC2 (S394, ab75602); Histone Deacetylase 1 (HDAC1, ab109411); Histone Deacetylase 3 (HDAC3, ab32369); Histone Deacetylase 4 (HDAC4, ab235583); Histone Deacetylase 6 (HDAC6, ab133493); Histone Deacetylase 8 (HDAC8, ab187139); S6K1 (ab32529); p‐S6K1 (ab131459); p38 (ab170099); p‐p38 (ab195049); and p21 (ab109520). 5‐Aza‐2ʹ‐Deoxycytidine (5‐Aza‐dC, A3656), Ezh2 inhibitor II (5005610001), BAY‐598 (SML1603), CI‐994 (C0621), RGFP966 (SML1652), tasquinimod (SML2489), SAHA (SML0061), cycloheximide (CHX; 239763‐M), actinomycin D (Act‐D, SBR00013), MG132 (M7449), rapamycin (V900930), SB203580 (S8307), and anisomycin (A5862) were purchased from Sigma‐Aldrich. Santacruzamate A (SCA; HY‐N0931), PCI34051 (HY‐15224), and CAY10603 (HY‐18613) were obtained from MedChemExpress.

### 2.4 Plasmids

The full‐length cDNAs of TRIB2, COP1, METTL14, DICER, ubiquitin, and Akt were amplified by RT‐PCR using total RNA from HEK293T cells. Myc‐ubiquitin, Myc‐TRIB2, Myc‐METTL14, and Myc‐COP1 were subcloned into a pRK5 vector with a Myc‐tag. Flag‐METTL14 and Flag‐TRIB2 were subcloned into a pRK5 vector with a Flag‐tag. V5‐COP1 was subcloned into a pRK5 vector with a V5‐tag. His‐ubiquitin was subcloned into a pcDNA3.1/His A vector (Invitrogen) with a His‐tag. TRIB2 VPM mutants were generated by site‐directed mutagenesis. For the generation of lentivirus‐based expression constructs, full‐length cDNAs for TRIB2, COP1, METTL14, DICER, and Akt were subcloned into the pLVX‐Puro vector (Takara Bio). Oligonucleotides targeting TRIB2 and METTL14 (Table S8) were synthesized and inserted into the short hairpin RNA (shRNA) expression vector pLVX‐shRNA (Takara Bio).

### 2.5 Establishment of patient‐derived xenograft (PDX) tumors

Fresh PDX tumor samples collected from two established PDX models (PDX #07 with high TRIB2 expression and PDX #12 with low TRIB2, passages three to four) were minced and subcutaneously implanted into the flanks of 3– to 4‐week‐old female BALB/c nude mice (Jiesijie Laboratory Animals). After the volume of the xenograft tumors reached ∼100 mm^3^, mice from each PDX model were randomly divided into two groups (five mice each): vehicle + ionizing radiation (IR, 10 Gy total); and SCA + IR (10 Gy total). SCA (20 mg/kg in dimethyl sulfoxide, DMSO) and vehicle (normal saline) were administered daily for 28 consecutive days by intraperitoneal injection. IR was performed using a Cs^137^ irradiator; a 5‐Gy dose was given on the seventh and 14th days after the mice were separated into groups. The tumor volume was measured every 5 days using a caliper and was determined as follows: length × width^2^ × 0.5. The mice were sacrificed 28 days after being separated into groups, and tumor samples were harvested. The tumor volumes were evaluated by researchers who were blinded to the initial treatment.

The in vivo study was approved by the Animal Care and Use Committee of Shanghai Tongji Hospital and conducted in accordance with ethical standards.

### 2.6 Protein stability

CHX (100 μg/ml) was added to the cells. After incubation for the indicated time periods (0, 3, or 12 h), the cells were harvested, and METTL14 expression was determined by western blotting.

### 2.7 RNA stability

Act‐D (5 μg/ml) was added to the cells. After incubation for the indicated time periods (0, 8, 24, or 48 h), the cells were harvested, and RNA was extracted and used for RT‐PCR.

### 2.8 Luciferase reporter assay

Fragments of the wild‐type (WT) TRIB2 3′ UTR (WT 3′ UTR) and mutant‐type (Mut) TRIB2 3′ UTR (Mut 3′ UTR) were synthesized and subcloned into the pmiRGLO3 vector (Promega) to create the pmiRGLO‐WT‐3′ UTR and pmiRGLO‐Mut‐3′ UTR vectors, respectively. These constructs were transfected into the indicated ESCC cells, which were also transfected with miR‐99a‐5p mimics, miR‐99a‐5p inhibitor, or control constructs. For the determination of promoter activity of pri‐mir‐99a, deletion mutants starting at positions −4000, −2500, −1500, −1000, or −500 nt for pri‐mir‐99a relative to the transcription start site (TSS) were amplified and subcloned in frame with a luciferase reporter gene in the vector pGL4.17 (Promega). The luciferase activity was measured with a Dual‐Glo Luciferase Assay System (Promega) and an illuminometer. Renilla luciferase was used as a control to normalize firefly luciferase intensity.

### 2.9 IHC and ISH

For IHC, paraffin‐embedded sections were deparaffinized and rehydrated and then subjected to antigen retrieval by boiling in citrate buffer (pH 6.0). Endogenous peroxidase activity was then blocked by incubating the sections in 3% H_2_O_2_. The sections were subsequently blocked with 10% goat serum and incubated with primary antibodies against TRIB2, ALDH1, SOX2, HDAC2, p21, Ki‐67, and METTL14 at 4°C overnight. After incubation of the sections with an Horseradish Peroxidase (HRP)‐conjugated secondary antibody, immunoreactive proteins were detected by 3,3′‐diaminobenzidine staining (Zhongshan Goldenbridge Biotechnology Company).

For ISH, the deparaffinized and rehydrated sections were subjected to nucleic acid retrieval followed by protease treatment. After incubation with Exiqon hybridization buffer (Exiqon), the sections were hybridized to fluorescein‐labeled miRNA probes for miR‐99a‐5p at 42°C for 2 h. The slides were then washed to remove nonspecific probes, followed by the sequential addition of anti‐fluorescein antibody and poly‐HRP to detect miRNA expression.

Counterstaining was performed with hematoxylin. The expression of target protein/miRNA was scored semiquantitatively (high or low using the staining index; SI) based on the product of staining intensity (scaled from 1 to 4) and the proportion of positively stained cells (0, no positive cells; 1, < 10%; 2, 10%–35%; 3, 35%–75%; 4, > 75% positive). Two pathologists scored each sample to avoid any bias. SI ≥ 8 was considered high expression, and SI < 8 was considered low expression.

### 2.10 Mass spectrum （MS） analysis

The total proteins were separated by sodium dodecyl sulfate polyacrylamide gel electrophoresis (SDS‐PAGE), stained with Coomassie blue, and the protein bands were collected by excision. The excised gel bands were minced, destained, and digested in sequencing‐grade trypsin. After digestion, the samples were desalted in preparation for MS analysis by a QExactive system (Thermo Fisher Scientific). Finally, Proteome Discoverer software (Thermo Fisher Scientific) was applied for protein identification and quantification.

### 2.11 5‐Ethynyl‐2′‐deoxyuridine (EdU) assay

Cell proliferation was assayed using an EdU assay kit (RiboBio) according to the manufacturer's instructions. In brief, ESCC cells were plated in 96‐well plates and incubated with EdU (50 μM) for 2 h at room temperature. The cells were then subjected to fixation, permeabilization, and EdU staining. Nuclei were counterstained with 4',6‐diamidino‐2‐phenylindole (DAPI, Sigma‐Aldrich). The proportion of EdU‐positive cells was assessed via fluorescence microscopy (Olympus).

### 2.12 Colony‐formation assay

The ESCC cells were seeded in 6‐well plates at a density of 300 cells/well. After attachment, the cells were irradiated at a single dose of 10 Gy and cultured for 3 weeks. The cells were then fixed in 4% paraformaldehyde, stained with 0.5% crystal violet, imaged, and counted.

### 2.13 Sphere‐formation assay

For the tumor sphere‐formation assay, ESCC cells were seeded in ultralow attachment 6‐well plates (Gibco) and cultured in serum‐free DMEM/F12 (Gibco) supplemented with basic fibroblast growth factor (10 ng/ml, Invitrogen) and epidermal growth factor (10 ng/ml, Invitrogen) for 14 days. The number and diameter of spheres were assessed under a light microscope (Olympus).

### 2.14 Comet assay

The alkaline comet assay was performed using a comet assay kit (ab238544, Abcam). In brief, ESCC cells were loaded onto comet slides covered with comet agarose. The cells were treated with lysis buffer and alkaline solution, and electrophoresis was conducted under alkaline conditions. Finally, the cells were fixed on the slide and stained with SYBR Green. Images of the cells were acquired by fluorescence microscopy (Olympus). CASP software (http://casplab.com) was used to analyze the percentage of DNA in the tail.

### 2.15 Caspase‐3 activity assay

Caspase‐3 activity was detected using a caspase‐3 activity assay kit (Beyotime) according to the manufacturer's instructions. Briefly, the cells were lysed in lysis detection solution and Ac‐DEVD‐pNA (2 mM) was then added and incubated for 2 h. The absorbance of each well was measured at 405 nm.

### 2.16 Immunofluorescence assay

For Ki‐67 staining, cells were washed with phosphate buffered saline (PBS), fixed in 4% paraformaldehyde, permeabilized with 0.1% Triton X‐100, and blocked with 3% bovine serum albumin (BSA). They were then incubated with primary antibodies against γ‐H2AX and then with a fluorescein isothiocyanate (FITC)‐conjugated secondary antibody (ab6717, Abcam). Nuclei were counterstained with DAPI (Sigma‐Aldrich). Images were acquired by fluorescence microscopy (Olympus).

### 2.17 Magnetic cell sorting

ESCC cells were preincubated with biotin‐conjugated anti‐CD90 (Abcam) or biotin‐conjugated anti‐CD271 (BD Biosciences) and then incubated with goat anti‐mouse IgG microbeads (Miltenyi Biotec). The labeled cells were then sorted using MACS LS columns (Miltenyi Biotec) according to the manufacturer's instructions.

### 2.18 Flow cytometry

To determine the CD90^+^ and CD271^+^ subpopulations, ESCC cells were stained with P‐phycoerythrin (PE)‐conjugated anti‐CD90 or PE‐conjugated anti‐CD271 (BioLegend) and subjected to CytoFLEX flow cytometry (Beckman Coulter). To assess ALDH1 activity, the cells were stained using the ALDEFLUOR™ detection kit (StemCell Technologies) according to the manufacturer's instructions and analyzed on a CytoFLEX flow cytometer. To assess cellular apoptosis, cells were double‐stained with annexin V‐FITC and propidium iodide (BD Biosciences) according to the manufacturer's instructions, and staining was detected using a CytoFLEX flow cytometer. All data were analyzed and plotted using FlowJo (TreeStar).

### 2.19 Subcellular fractionation of RNA

NE‐PER nuclear and cytoplasmic extraction reagents (Pierce) were used to separate the nuclear and cytoplasmic fractions of the indicated cells. RNA was extracted from each fraction using TRIzol and quantified by RT‐PCR.

### 2.20 Polymerase Chain Reaction （PCR） of extracted RNA

Total RNA was extracted using TRIzol (Invitrogen) and reverse‐transcribed to cDNA using the PrimeScript RT reagent kit (Takara Bio). SYBR Premix Ex Taq (Takara Bio) was employed to identify the levels of the corresponding RNAs. The results were normalized to those obtained using GAPDH. The mRNA levels were calculated using the 2^–ΔΔCt^ method.

To measure miRNA expression, the miRVana™ miRNA isolation kit (Ambion) was used to extract small RNAs from the samples. The extracted small RNAs were labeled with poly(A) and subjected to cDNA synthesis and RT‐PCR using the QuantiMir™ RT kit (System Biosciences). 5S rRNA was used as a control for normalization.

The TSS of pri‐mir‐99a was determined using the SMART RACE cDNA amplification kit (Clontech). Briefly, the extracted RNA was reverse‐transcribed using a PowerScript RT kit (Clontech). Gene‐specific primers (GSP) and a Universal primer (Clontech) were used to conduct nested PCR. The PCR products were separated by agarose gel electrophoresis, purified and inserted into T‐Vector pMD19 (TaKaRa) for sequencing. The primers used for detection are shown in Table S8.

### 2.21 Cell transfection

For lentivirus‐based transduction, concentrated viral particles were transfected into ESCC cells for 24 h in medium containing polybrene (Sigma‐Aldrich). The supernatant was replaced with a complete culture medium, and the transfected cells were selected by culturing in the presence of puromycin (Sigma‐Aldrich) for 1 week.

small interfering RNAs (siRNAs) targeting DGCR8, COP1, Akt and nonspecific siRNA and human miR‐99a‐5p mimics, miR‐99a‐5p inhibitor, and the corresponding controls were synthesized by RuiBoBio. Lipofectamine 3000 (Invitrogen) was used to transfect cells with siRNA, miRNA, and miRNA inhibitors.

The pRK5‐ and pcDNA3.1‐based plasmids were transfected into ESCC and 293T cells using Lipofectamine 3000 (Invitrogen) according to the manufacturer's instructions.

### 2.22 Western blotting, immunoprecipitation (IP) and chromatin IP (ChIP)‐PCR assay

Total protein was extracted from tissues and cells using radio‐immunoprecipitation assay (RIPA) lysis buffer (Solarbio) supplemented with protease and phosphatase inhibitors. The concentration of the extracted proteins was determined using the bicinchoninic acid method (Solarbio).

For western blotting, the extracted proteins were separated by 10% SDS‐PAGE and transferred to poly(vinylidene fluoride) (PVDF) membranes (Invitrogen). The membranes were blocked in blocking buffer (EpiZyme) and incubated with the indicated primary antibodies overnight at 4°C. The membranes were then incubated with HRP‐conjugated secondary antibodies (Abcam) for 1 h at room temperature and washed with tris‐buffered saline and tween 20 (TBST). The blots were developed via the enhanced chemiluminescence method (EpiZyme).

For IP, the cells were treated as described above. The extracts were precleared using protein A‐agarose, and IP was performed by incubating the supernatants with primary antibodies against METTL14, TRIB2, Myc‐tag, Flag‐tag, and V5‐tag for 1 h at 4°C. The samples were then incubated with protein A/G overnight at 4°C. After centrifugation, the protein A‐agarose‐antigen‐antibody complexes were washed with lysis buffer and resuspended in loading buffer (Solarbio). The proteins were separated by SDS‐PAGE and detected by western blotting. IP with rabbit IgG isotype was conducted as a negative control.

The ChIP‐PCR assay was conducted according to the instructions provided with the ChIP assay kit (Millipore). Briefly, the cells were treated as described above, crosslinked with 1% formaldehyde, washed, lysed in ChIP lysis buffer, and sonicated. The samples were then diluted with ChIP dilution buffer, precleared with protein A/agarose/salmon sperm DNA and subjected to IP with primary antibodies against H3K9Ac, H3K14Ac, and histone H3 for 1 h at 4°C. The samples were then incubated with protein A/G overnight at 4°C. The immune complexes were sequentially washed and mixed with elution buffer to reverse the crosslinking. The genomic region of the p21 promoter was then amplified by RT‐PCR. Fold enrichment of H3K9Ac, H3K14Ac, and H3 was expressed as the observed fold change over the input: 2^Ct (input) – Ct (ChIP)^.

### 2.23 Ubiquitination assay

The cells were transfected with various constructs together with Myc‐ubiquitin and Flag‐METTL14, treated with MG132, and lysed in RIPA lysis buffer. Ubiquitination was assessed by IP with an antibody against Flag‐tag and subsequent western blotting with an anti‐Myc Ab. Alternatively, the cells were transfected with His‐ubiquitin and Flag‐METTL14, treated with MG132, lysed in denaturing buffer (6 M guanidine‐HCl, 0.1 M Na_2_HPO_4_/NaH_2_PO_4_,^11^ and 10 mM imidazole), followed by incubation with Ni‐nitrilotriacetic acid (Ni‐NTA) agarose. The beads were washed and then assessed by western blotting.

### 2.24 m^6^A‐RT‐PCR

Total RNA was extracted using TRIzol (Invitrogen). Fifty nanograms of total RNA were removed as an input control, and an anti‐m^6^A antibody (Abcam) was added to the remaining RNA in buffer containing 150 mM NaCl, 0.1% NP‐40, 10 mM Tris, and RNase inhibitor. The m^6^A pull‐down portion was collected via IP with Dynabeads^®^ Protein A (Invitrogen), washed with elution buffer, and recovered by ethanol precipitation. The m^6^A pull‐down RNA was then amplified by RT‐PCR.

### 2.25 Viability assay

Cell viability assays were conducted using Cell Counting Kit‐8 (CCK‐8) (Dojindo) according to the manufacturer's instructions. In brief, ESCC cells were seeded in 96‐well plates at a density of 5000 cells/well. After attachment, the cells were incubated with CCK‐8 reagent at 37°C for 2 h. Cell viability was indicated by the absorbance at 450 nm.

### 2.26 Bioinformatics

The Gene Expression Omnibus (GEO) dataset (https://www.ncbi.nlm.nih.gov/geo/) (GSE43732, GSE55856, GSE66274, and GSE114110) was used to analyze the differentially expressed miRNAs in ESCC. GSE44021 was used to analyze the differentially expressed mRNAs in ESCC. The ESCC dataset in The Cancer Genome Atlas (TCGA) database (https://portal.gdc.cancer.gov/) was used to analyze the prognostic value of miR‐99a‐5p in ESCC and the correlation between miR‐99a‐5p and TRIB2, ALDH1, SOX2, and METTL14. StarBase (http://starbase.sysu.edu.cn/) was used to predict the mRNAs targeted by miR‐99a‐5p. Raw data were collected from these datasets and manipulated, analyzed, and presented via R 3.6.0 and GraphPad Prism 7.0.

All code supporting the conclusions of the study is available from the corresponding author by reasonable request.

### 2.27 Statistical analysis

The data are presented as the mean ± SD. Data analysis was conducted using GraphPad Prism 7.0. Unpaired or paired two‐tailed Student's *t*‐tests were conducted for comparisons between two groups. One‐way analysis of variance (ANOVA) with Tukey's post hoc test was performed to compare more than two groups. Differences among variables were assessed by Fisher's exact test. Correlations between the expression levels of different factors were assessed by Pearson correlation coefficient and Spearman correlation coefficient analyses. The survival data were analyzed using the log‐rank test and presented as Kaplan–Meier (K‐M) survival curves. A Cox proportional hazards model was employed to perform univariate and multivariate analyses. All experiments were repeated at least three times. All data were tested beforehand to meet the assumptions of the statistical analysis (e.g., normal distribution, adequate statistical power, and homogeneity of variance). All experiments were repeated at least three times. Differences with *p* < 0.05 were considered significant.

## 3 RESULTS

### 3.1 miR‐99a‐5p is downregulated in ESCC CSCs and predicts unfavorable clinical outcome in ESCC patients

**FIGURE 1:**
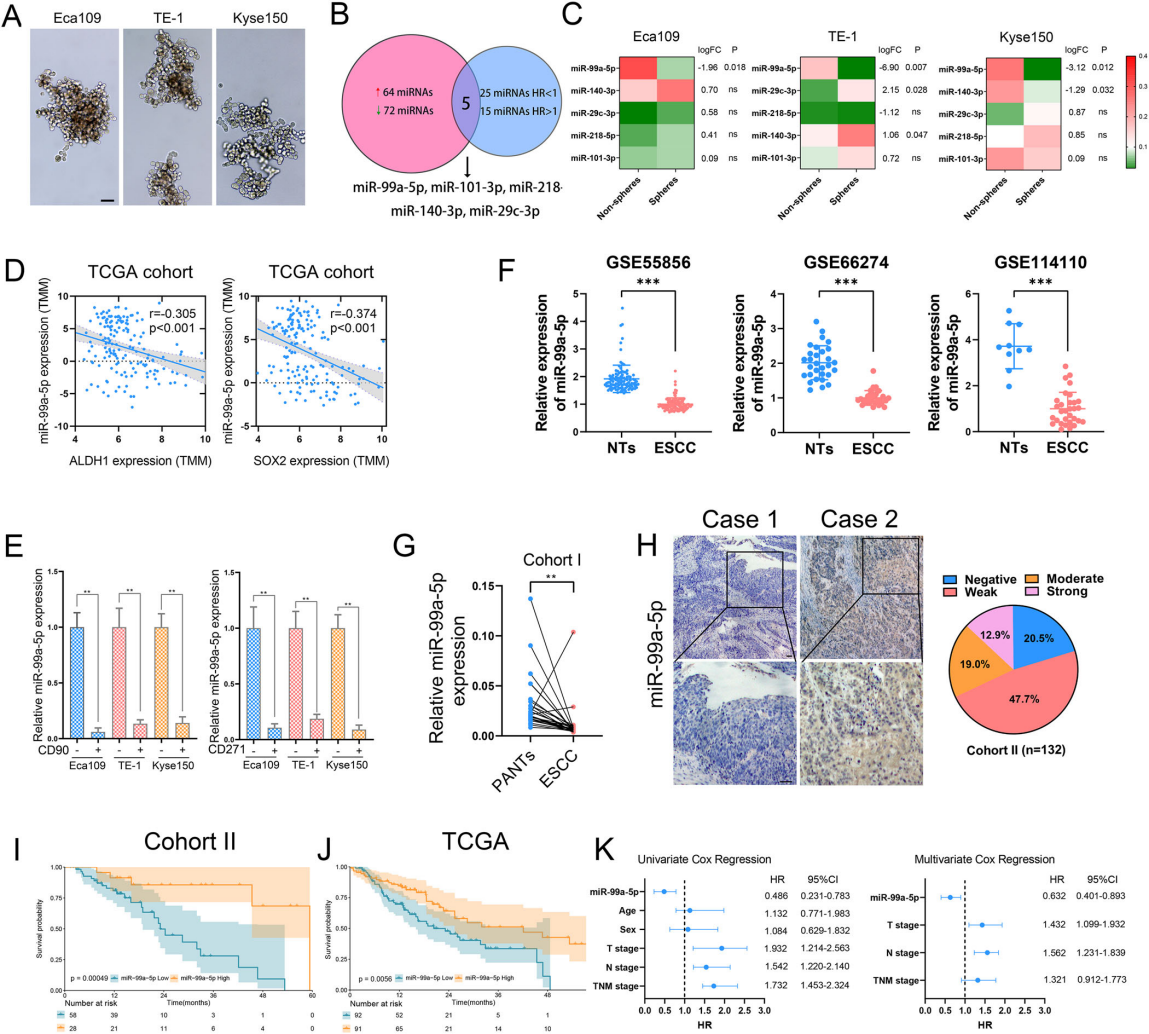
miR‐99a‐5p is downregulated in esophageal squamous cell carcinoma (ESCC), and its expression correlates closely with cancer stem‐like cell (CSC) properties of ESCC (A) Representative images of spheres of indicated ESCC cells enriched by serially replating in ultralow attachment plates. Scale bars: 100 μm. (B) Venn diagram showing overlap microRNAs (miRNAs) that both dysregulated and are survival‐predictable in ESCC. (C) Heatmap showing the expression of five overlapped miRNAs relative to 5S rRNA as determined by multiplex quantitative real‐time PCR (qRT‐PCR). Log fold change represents log_2_ fold change of miRNA expression between spheres and non‐spheres. (D) Correlation between miR‐99a‐5p and ALDH1 (left) and SOX2 (right) mRNA levels in patients in the The Cancer Genome Atlas (TCGA) dataset. (E) Cells were first sorted based on the expression of CD90^+^ and CD271^+^, then, miR‐99a‐5p expression in CD90^+^ (left) and CD271^+^ (right)‐enriched cell populations was detected by qRT‐PCR. (F) Expression profile of miR‐99a‐5p in ESCC tissues and nontumorous tissues derived from the Gene Expression Omnibus dataset. NTs, Non‐tumorous tissues. (G) miR‐99a‐5p expression in 26 paired ESCC tissues and PANTs was measured by qRT‐PCR; 5S rRNA was used as an internal control. PANTs, paired adjacent non‐tumorous tissues. (H) Representative images of in situ hybridization (ISH) staining for miR‐99a‐5p expression in ESCC tissues. Negative (staining index (SI) < 3), weak (3 < SI < 6), moderate (6 < SI < 10), strong (SI > 10). Scale bars: 100 μm. (I‐J) Kaplan–Meier overall survival (OS) analysis of miR‐99a‐5p expression in ESCC tissues in Cohort II (left) and from patients in the TCGA cohort (right). (K) Univariate and multivariate Cox regression analyses showed that miR‐99a‐5p was an independent prognostic factor in ESCC. The data represent the mean ± SD. *p < 0.05, **p < 0.01, ***p < 0.001. *p‐*values were determined by unpaired Student's *t*‐test (E and F), paired Student's *t*‐test (G), and log‐rank test (I and J). The correlation was determined by the Pearson correlation test (D)

To examine possible miRNAs involved in mediating CSC properties in ESCC, we first collected spheres of three ESCC cell lines (Eca109, Kyse150, and TE‐1) by serially replating in ultralow attachment plates (Figure 1A); the stemness of these spheres was identified by measuring several CSC markers (ALDH1, SOX2, NANOG, OCT4, BMI‐1, ABCG2, CD90 and CD271) (Figure S1A–C). Next, we analyzed the expression of miRNAs in the GEO dataset (GSE43732) and identified 136 miRNAs that were dysregulated in ESCC (Table S1). By conducting Cox regression analysis for data deposited in the TCGA, we screened 40 prognostic significant miRNAs (Table S2). Finally, we focused on the five miRNAs that were dysregulated and survival‐predictable in ESCC and measured their expression in spheres and non‐spheres of ESCC cells by quantitative RT‐PCR (Figure 1B). Among these miRNAs, miR‐99a‐5p had the most impact on CSC properties in ESCC cells and was consistently decreased in the spheres of three ESCC cell lines (Figure 1C). Co‐expression analysis further revealed that miR‐99a‐5p negatively correlated with ALDH1 and SOX2, two CSC markers in ESCC (Figure 1D). Moreover, miR‐99a‐5p was dramatically decreased in CD90^+^‐ and CD271^+^‐enriched subpopulations of ESCC cells (Figure 1E). All of these findings indicated a potential role of miR‐99a‐5p in regulating CSC properties of ESCC.

Validation from the external dataset (GSE55856, GSE66274, and GSE114110) and Cohort I in our hospital further corroborated that miR‐99a‐5p had significantly reduced expressed in ESCC (Figure 1F,G). ISH also showed that the abundance of miR‐99a‐5p was low in most paraffin‐embedded ESCC tissues detected (Figure 1H); qPCR analysis confirmed the inverse relationship between miR‐99a‐5p and CD90 in ESCC tissues in Cohort I. High miR‐99a‐5p expression was shown to significantly associate with favorable overall survival (OS) in ESCC patients, which was consistent with TCGA (Figure 1I,J). Univariate and multivariate Cox regression analyses also indicated miR‐99a‐5p expression as an independent prognostic factor for OS (Figure 1K).

Taken together, these results suggested that downregulated miR‐99a‐5p may promote CSCs persistence and lead to a poor clinical outcome of ESCC.

### 3.2 miR‐99a‐5p inhibits CSC persistence and sensitizes ESCC cells to radiotherapy

**FIGURE 2:**
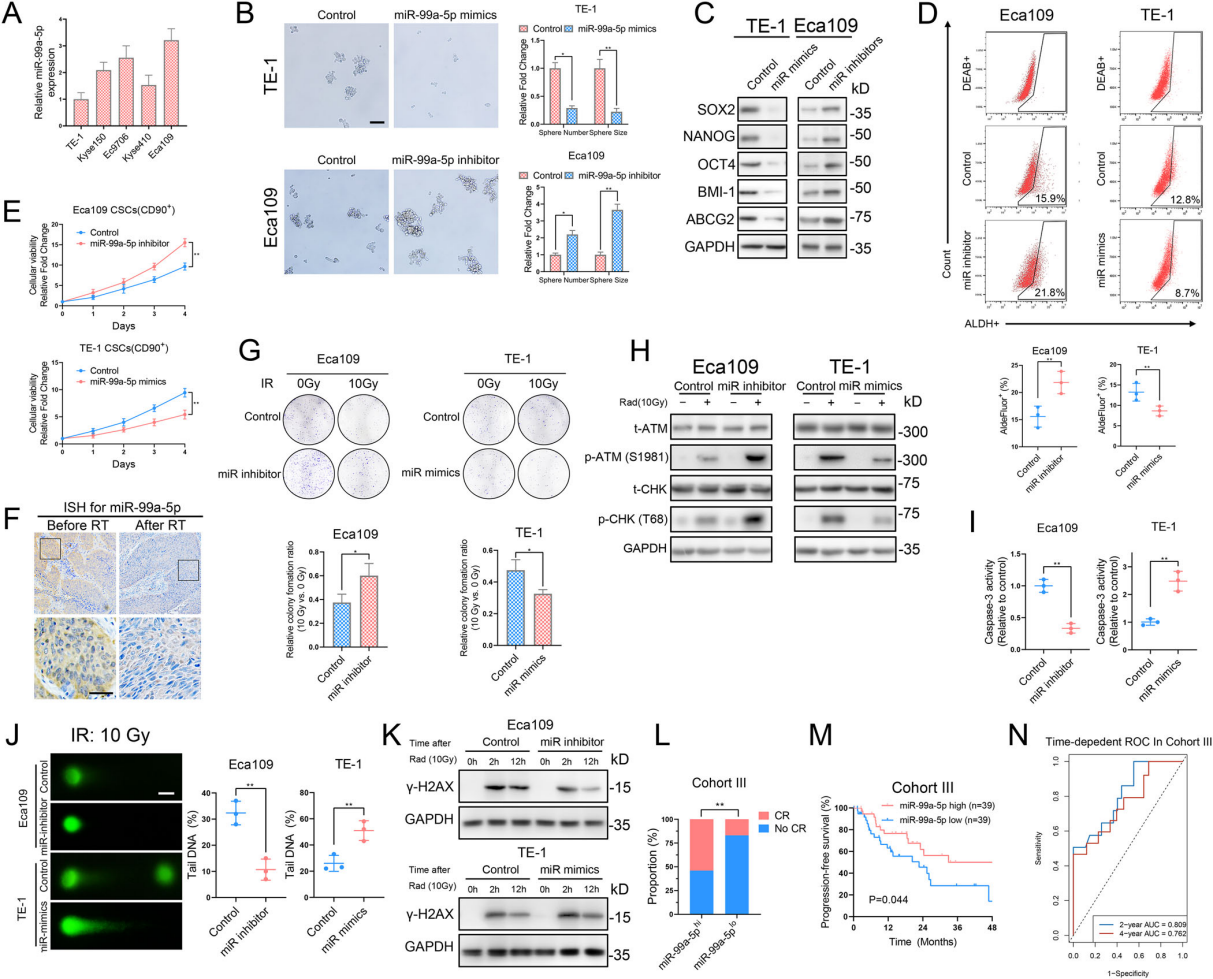
miR‐99a‐5p suppresses CSC properties and radioresistance of ESCC cells (A) Relative expression of miR‐99a‐5p in ESCC cell lines (Eca109, Ec9706, Kyse410, Kyse150, and TE‐1) as detected by qRT‐PCR. (B) Representative images (left) and statistical quantification (right) of sphere formation by the indicated ESCC cells. Scale bars: 100 μm. (C) Western blotting analysis of CSC markers in the indicated ESCC cells. (D) Representative images (upper) and statistical quantitation (lower) of the results of flow cytometric analysis for determination of ALDH activity in the indicated ESCC cells. (E) Cell viability assay of the CD90^+^‐enriched cell population in the indicated ESCC cells. (F) Representative images showing ISH staining of miR‐99a‐5p in ESCC tissues from patients at Tongji Hospital before and after radiotherapy. Scale bars: 100 μm. (G) Representative images (upper) and statistical quantification (lower) showing colony formation by the indicated ESCC cells after ionizing radiation (IR) at a dose of 10 Gy. (H) Western blotting analysis of the total and phosphorylated proportion of checkpoint markers ATM and CHK in the indicated ESCC cells 4 h after IR at a dose of 10 Gy. (I) Relative caspase‐3 activity in the indicated ESCC cells 24 h after IR at a dose of 10 Gy. (J) Representative images (left) and statistical quantification (right) of the comet assay results in the indicated ESCC cells 6 h after IR at a dose of 10 Gy. Scale bars: 10 μm. (K) After IR at a dose of 10 Gy, the indicated ESCC cells were cultured under normal conditions for 2 and 12 h, and γ‐H2AX expression in each group was determined by western blotting. (L) Patients with higher miR‐99a‐5p expression were more likely to respond well to radiotherapy than those with low miR‐99a‐5p expression. (M) K‐M plot showing the relationship between miR‐99a‐5p levels and patient progression‐free survival (PFS) in Cohort III. (N) The time‐dependent ROC analysis of patients in Cohort III revealed that miR‐99a‐5p was an independent factor for predicting 2‐ and 4‐year PFS. The data represent the mean ± SD. *p < 0.05, **p < 0.01, ***p < 0.001. *p‐*values were determined by unpaired Student's *t*‐test (B, D, E, G, I, and J), the log‐rank test (M), and Fisher's exact test (L)

We then evaluated the potential function of miR‐99a‐5p on the stemness of ESCC cells. We first validated the expression of miR‐99a‐5p among several ESCC cell lines (Figure 2A). We overexpressed miR‐99a‐5p by miRNA mimics in TE‐1 cells (lowest miR‐99a‐5p level) and downregulated miR‐99a‐5p expression via a miRNA inhibitor in Eca109 cells (highest miR‐99a‐5p level). In vitro assays showed that sphere formation was significantly enhanced in miR‐99a‐5p‐downregulated ESCC cells but was reduced following miR‐99a‐5p overexpression (Figure 2B). Further, miR‐99a‐5p overexpression decreased, whereas miR‐99a‐5p downregulation promoted, the expression of CSC markers as well as the proportions of CD90^+^ and CD271^+^ cells (Figures 2C,D and S2A–C). Finally, miR‐99a‐5p inhibition in ESCC CSCs increased the proliferation rate, while miR‐99a‐5p overexpression exhibited an opposite effect (Figure 2E). However, overexpression or inhibition of miR‐99a‐5p had no effect on the proliferation of non‐stem ESCC cells (Figure S2D,E).

The stemness of cancer cells has been well established to be associated with the induction of radioresistance.^10^ In ESCC tissues derived from patients who received radiotherapy, miR‐99a‐5p was significantly lower after treatment with radiotherapy than before treatment (Figures 2F and S2F). To further investigate whether downregulated miR‐99a‐5p participated in the radioresistance of ESCC, we evaluated the colony‐formation ability of ESCC cells after IR. We found that miR‐99a‐5p overexpression significantly inhibited colony formation and that miR‐99a‐5p inhibition had the opposite effect (Figure 2G). It has been shown that the induction of DNA damage is the primary mechanism by which IR kills cancer cells.^12^ Consistently, phosphorylation of the DNA damage checkpoint response markers CHK2 and ATM was dramatically induced in miR‐99a‐5p‐depleted cells but decreased in miR‐99a‐5p‐overexpression cells (Figure 2H). miR‐99a‐5p also induced the apoptosis of ESCC cells as indicated by the change in caspase‐3 expression after IR (Figure 2I). Moreover, comet assays and evaluation of the time course of changes in γ‐H2AX expression confirmed that miR‐99a‐5p overexpression compromised the DNA repair efficiency of ESCC cells, while miR‐99a‐5p inhibition had the opposite effect (Figures 2J,K and S2G). Again, miR‐99a‐5p did not impact the apoptosis or DNA repair ability of non‐stem ESCC cells (Figure S2H–J).

We retrospectively analyzed an enlarged radiotherapy cohort (Cohort III) containing 78 ESCC cases with locally advanced stage receiving radiotherapy. Those with high miR‐99a‐5p expression were more likely to achieve complete response (Figure 2L). Patients with lower miR‐99a‐5p expression had much worse progression‐free survival (PFS), compared with those with high miR‐99a‐5p expression levels (Figure 2M). By performing time‐dependent receiver operating characteristic (ROC) analysis according to the level of miR‐99a‐5p in Cohort III, we found that the expression of miR‐99a‐5p in ESCC tissues exhibited a satisfactory predictive performance for increasing survival risks in terms of PFS in ESCC (Figure 2N). These observations further confirmed the inhibitory roles of miR‐99a‐5p on CSC properties and the radioresistance of ESCC cells.

### 3.3 Downregulation of miR‐99a‐5p is due to the decreased activity of the m^6^A writer METTL14 in ESCC

**FIGURE 3:**
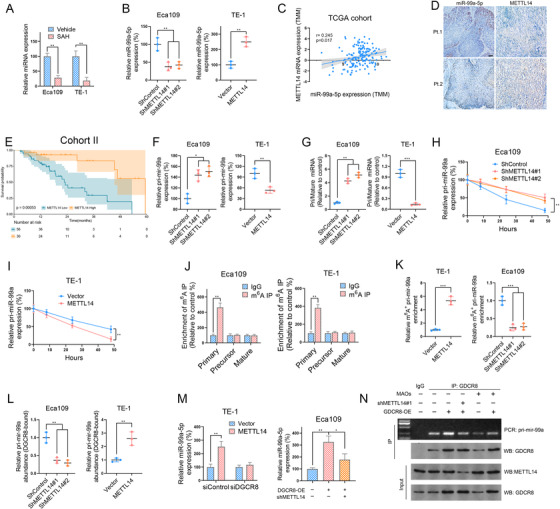
Methyltransferase‐like 14 (METTL14)‐mediated methyladenosine (m^6^A) methylation m^6^A modification regulates miR‐99a‐5p expression in ESCC (A) miR‐99a‐5p expression in Eca109 and TE‐1 cells after treatment with or without S‐adenosylhomocysteine (5 μM) was measured by RT‐PCR. (B) METTL14 overexpression increased, whereas METTL14 knockdown decreased, miR‐99a‐5p expression in ESCC cells as measured by RT‐PCR. (C) Correlation between miR‐99a‐5p and METTL14 mRNA levels in ESCC tissues from patients in the TCGA dataset. (D) Immunohistochemistry (IHC) and ISH showing that METTL14 expression positively correlated with the miR‐99a‐5p level in ESCC tissues obtained from patients at Tongji Hospital. Scale bars: 50 μm. (E) Kaplan–Meier OS analysis of METTL14 expression in ESCC tissues obtained from patients at Tongji Hospital. (F) METTL14 overexpression reduced, whereas METTL14 silencing upregulated, pri‐mir‐99a expression in ESCC cells as detected by RT‐PCR. (G) Ratio of pri‐mir‐99a expression to mature miR‐99a‐5p expression in ESCC cells as determined by RT‐PCR. (H‐I) The indicated ESCC cells were treated with actinomycin D (5 μg/ml) for the indicated time periods, and the expression level of pri‐mir‐99a was measured by qRT‐PCR. (J) N^6^‐m^6^A‐RNA immunoprecipitation (MeRIP)‐qPCR analysis of primary, precursor, and mature miR‐99a‐5p in Eca109 and TE‐1 cells. (K) MeRIP‐qPCR analysis of pri‐mir‐99a after METTL14 knockdown or overexpression in the indicated ESCC cells. (L) The levels of DiGeorge critical region 8‐dependent (DGCR8)‐bound pri‐mir‐99a in the indicated ESCC cells after METTL14 knockdown or overexpression were determined by IP of DGCR8‐associated RNA followed by RT‐PCR. (M) The expression of mature miR‐99a‐5p in ESCC cells transfected with the indicated constructs was determined by qPCR. (N) RIP assays for detecting the interaction between DGCR8 and pri‐mir‐99a. Bottom, IP efficiency of the DGCR8 antibody. Top, the abundance of DGCR8‐associated pri‐mir‐99a was analyzed by qPCR and normalized to the input control. The data are presented as the mean ± SD. *p < 0.05, **p < 0.01, ***p < 0.001. *p‐*values were determined by one‐way ANOVA with Tukey's post hoc test (B, F, G, H, K, L, and M), unpaired Student's *t*‐test (A, B, F, G, I, J, K, L, and M) or the log‐rank test (E). Correlations were determined by the Pearson correlation test (C)

To explore whether the mechanism behind the downregulated miR‐99a‐5p expression in CSCs is transcriptional or post‐transcriptional, we identified the TSS and promoter region of pri‐mir‐99a by 5‘ rapid‐amplification of cDNA end (5′ RACE) and luciferase assays, respectively (Figure S3A–C). Then, luciferase reporter plasmids containing the putative pri‐mir‐99a promoter were transfected into spheres and nonspheres of ESCC cells. Our results showed that a similar promoter activity was observed in the spheres and nonspheres of ESCC cells. In addition, we found no overt alterations in miR‐99a‐5p expression following treatment with HDAC inhibitors, ruling out the possibility that miR‐99a‐5p expression is regulated by histone acetylation (Figure S3E). Ultimately, the Act assay showed that miR‐99a‐5p was more stable, while pri‐mir‐99a decayed faster in nonspheres of ESCC cells (Figure S3F). These results indicated that the low expression of miR‐99a‐5p in CSCs was due to post‐transcriptional effects but not transcriptional effects.

m^6^A modification has been reported to regulate the expression and biogenesis of miRNAs at the posttranscriptional level.^13^ Using SRAMP, we found two m^6^A sites with high confidence along the full sequence of pri‐mir‐99a (Figure S3G). Consistently, we found that S‐adenosylhomocysteine (SAH), a METTL3/METTL14 dual inhibitor, greatly suppressed miR‐99a‐5p expression in ESCC cells (Figure 3A). We further silenced METTL3 and METTL14 and found that only METTL14 knockdown, but not METTL3, decreased miR‐99a‐5p expression in Eca109 cells (Figures 3B and S3H–J). Conversely, METTL14 overexpression in TE‐1 cells significantly increased miR‐99a‐5p expression (Figures 3B and S3K). Moreover, miR‐99a‐5p and METTL14 were highly coexpressed in ESCC tissues as illustrated by the TCGA cohort and tissues from patients in our hospital (Figure 3C,D). Survival analysis revealed that low METTL14 abundance predicted unfavorable OS in ESCC patients (Figure 3E).

Intriguingly, although METTL14 did not influence the promoter activity of primary mir‐99a (Figure S3L), pri‐mir‐99a expression and the ratio of pri‐mir‐99a/mature miR‐99a‐5p were increased in the METTL14‐silenced ESCC cells but reduced in the METTL14‐overexpressing ESCC cells (Figure 3F,G). Importantly, METTL14 did not influence the distribution of pre‐mir‐99a among different cellular compartments (Figure S4A). We surmised that pri‐mir‐99a might undergo m^6^A modification and then be further processed by the microprocessor complex subunit DGCR8 as illustrated in a recent study.^13^ To test our hypothesis, we measured the stability of primary and mature miR‐99a‐5p by treating cells with Act‐D. We found that METTL14 promoted the turnover of pri‐mir‐99a but stabilized its mature form in ESCC cells (Figures 3H,I and S4B). Furthermore, m^6^A‐RNA IP (MeRIP) qPCR revealed a significant enrichment of pri‐mir‐99a but not of precursor or mature miR‐99a‐5p occurred in the anti‐m^6^A‐antibody group (Figure 3J). METTL14 knockdown considerably decreased the level of m^6^A‐modified pri‐mir‐99a, and METTL14 overexpression markedly increased it (Figure 3K). Ultimately, a decrease in the amount of pri‐mir‐99a bound to DGCR8 was observed after METTL14 knockdown, whereas METTL14 overexpression resulted in the opposite effect (Figure 3L). DGCR8 overexpression in ESCC cells increased the expression of miR‐99a‐5p, and this effect was reversed by METTL14 knockdown. Conversely, DGCR8 knockdown completely blocked the METTL14‐mediated effect (Figures 3M and S4C). We designed a set of sequence‐specific morpholino antisense oligos targeting the m^6^A site in pri‐mir‐99a. The GDCR8 RIP we performed showed that DGCR8 overexpression promotes its ability to bind with pri‐mir‐99a. By contrast, either METTL14 silencing or blocking METTL14‐mediated m^6^A modification of pri‐mir‐99a strongly inhibited the interaction between GDCR8 and pri‐mir‐99a in cells with or without GDCR8 overexpression (Figures 3N and S4D). GDCR8 overexpression markedly decreased the level of pri‐mir‐99a, which was reversed by METTL14 silencing (Figure S4E).

Collectively, these data indicate that METTL14 enhances miR‐99a‐5p expression by modulating m^6^A‐dependent, DGCR8‐mediated pri‐mir‐99a processing.

### 3.4 miR‐99a‐5p downregulation relieves its repressive effect on TRIB2 expression in ESCC

**FIGURE 4:**
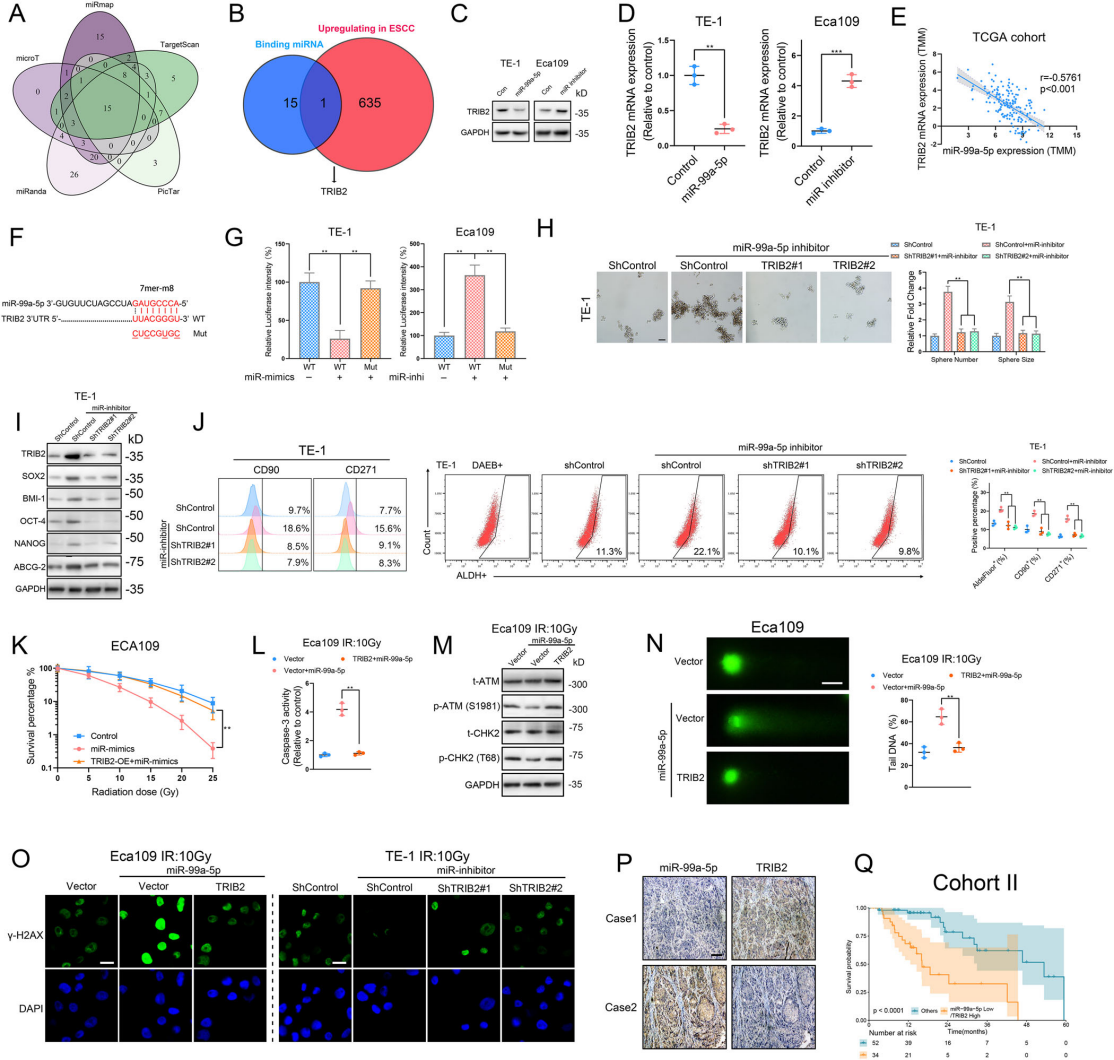
TRIB2 is the downstream target of miR‐99a‐5p, which promotes cancer cell stemness and radioresistance of ESCC (A) Venn diagram showing mRNAs that possesses miR‐99a‐5p binding sites in its 3′ untranslated region (3′ UTR) derived from the combined analysis of five web‐based databases (miRmap, microT, miRanda, PicTar, and TargetScan). mRNAs predicted to bind with miR‐99a‐5p that were common to all databases were selected as candidate mRNAs. (B) Venn diagram showing mRNAs that had both upregulated expression in ESCC and potential binding sites for miR‐99a‐5p. (C‐D) The indicated ESCC cells were transfected with control RNA, miR‐99a‐5p mimics, or miR‐99a‐5p inhibitor, and the protein and mRNA levels of TRIB2 were determined by western blotting (C) and RT‐PCR (D). (E) Correlation between tribble 2 (TRIB2) and miR‐99a‐5p expression in patients in the TCGA cohort. (F) Schematic representation of the miR‐99a‐5p‐binding region within the 3′ UTR of TRIB2 mRNA as predicted by starBase (http://starbase.sysu.edu.cn). (G) ESCC cells were cotransfected with control RNA, miR‐99a‐5p mimics, or miR‐99a‐5p inhibitor, and with the wild‐type (WT) 3′ UTR of TRIB2 or mutant‐type (Mut) TRIB2 3′ UTR. The firefly luciferase values were normalized to the intensity of Renilla luciferase. (H) Representative images (left) and statistical quantification (right) of sphere formation by the indicated ESCC cells. Scale bars: 100 μm. (I) Western blotting analysis of CSC markers in the indicated ESCC cells. (J) Representative images and statistical quantification of the results of flow cytometric analyses to determine the ALDH activity and the relative proportions of CD90‐positive and CD271‐positive subpopulations in the indicated ESCC cells. (K) Clonogenic assay of Eca109 cells transfected with the indicated construct after an IR dose of 10 Gy. The survival fraction was calculated as (number of colonies formed/number of cells plated)_irradiated_/(number of colonies formed/number of cells plated)_control_. (L) Relative caspase‐3 activity of the indicated ESCC cells 24 h after an IR dose of 10 Gy. (M) Western blotting analysis of the phosphorylated and total amounts of the checkpoint markers ATM and CHK in the indicated ESCC cells 4 h after an IR dose of 10 Gy. (N) Representative images (left) and statistical results (right) of the comet assay in the indicated ESCC cells 6 h after an IR dose of 10 Gy. Scale bars: 10 μm. (O) Immunofluorescence staining of γ‐H2AX in the indicated ESCC cells 12 h after an IR dose of 10 Gy. Scale bars: 10 μm. (P) Representative images showing IHC staining for TRIB2 and ISH detection of miR‐99a‐5p in ESCC patients at Tongji Hospital. Scale bars: 100 μm. (Q) Kaplan–Meier OS analysis of ESCC patients at Tongji Hospital with the corresponding expression profiles. The data are presented as the mean ± SD. *p < 0.05, **p < 0.01, ***p < 0.001. *p‐*values were determined by unpaired Student's *t‐*test (D), one‐way ANOVA with Tukey's post hoc test (G, H, J, K, L, and N), and the log‐rank test (Q). Correlations were determined by the Pearson correlation test (E)

To explore the direct downstream target of miR‐99a‐5p, we first integrated data from five databases (miRmap, microT, miRanda, PicTar, and TargetScan) and identified 15 candidate mRNAs containing potential binding sites of miR‐99a‐5p on its 3′ UTR (Figure 4A, Table S3). Furthermore, analysis of mRNA expression in the GSE44021 dataset identified 635 mRNAs that were upregulated in ESCC (Table S4). Finally, only TRIB2 both induced the upregulated expression in ESCC and possessed potential binding sites for miR‐99a‐5p (Figure 4B). Moreover, by transfecting cells with miRNA mimics or inhibitors, we showed that miR‐99a‐5p mimics decreased TRIB2 expression, whereas an miR‐99a‐5p inhibitor elevated it (Figure 4C and D). Co‐expression analysis comparing miR‐99a‐5p and TRIB2 levels reported in TCGA databases corroborated this finding (Figure 4E).

We next examined whether miR‐99a‐5p directly targets TRIB2 by cloning the WT 3′ UTR of TRIB2 and versions of the 3′ UTR with mutations in potential binding sites (Figure 4F). A luciferase assay demonstrated that miR‐99a‐5p significantly inhibited expression of the reporter gene associated with WT TRIB2 construct, while the Mut TRIB2 constructs fully blocked the miR‐99a‐5p‐mediated effect. In contrast, miR‐99a‐5p inhibition increased the luciferase activity of the WT construct, but no overt alteration in reporter activity was observed with the Mut constructs (Figure 4G).

We next explored whether the suppressive effect of miR‐99a‐5p on CSC phenotype is mediated by downregulating TRIB2. miR‐99a‐5p inhibition‐promoted CSC properties were attenuated by TRIB2 silencing as determined by sphere formation, ALDH1 activity, and CSC markers (Figure 4H–J). Conversely, the attenuation of CSC phenotypes caused by miR‐99a‐5p overexpression was completely rescued by TRIB2 overexpression (Figure S5A–D).

We further assessed the impact of miR‐99a‐5p/TRIB2 axis on radioresistance. As predicted, the colony‐formation ability was enhanced, and caspase‐3 activity was decreased, after miR‐99a‐5p depletion after IR, and these activities were rescued by TRIB2 knockdown (Figure S5E,F). Consistently, TRIB2 overexpression substantially restored the radioresistance of miR‐99a‐5p‐overexpressing cells (Figure 4K,L). Moreover, by analyzing DNA repair efficiency as determined by the DNA repair checkpoint response, comet assays, and γ‐H2AX expression, we found that Eca109 cells transfected with miR‐99a‐5p mimics displayed increased sensitivity to radiotherapy, compared with that of control cells and that of TRIB2/miR‐99a‐5p co‐expressing cells (Figure 4M–O). We made a similar observation in TE‐1 cells, in which the enhanced DNA damage repair through miR‐99a‐5p inhibition was completely reversed by TRIB2 silencing (Figures S5G–J and 4O).

Ultimately, the ISH revealed that low miR‐99a‐5p expression correlated with high TRIB2 expression (Figure 4P). A combination of low miR‐99a‐5p and high TRIB2 expression predicted poor OS (Figure 4Q).

Taken together, our findings demonstrated that the suppressive effect of miR‐99a‐5p on CSC persistence and radioresistance is through inhibiting TRIB2 expression.

### 3.5 TRIB2 suppresses METTL14 expression via COP1‐dependent ubiquitin‐mediated proteolysis

**FIGURE 5:**
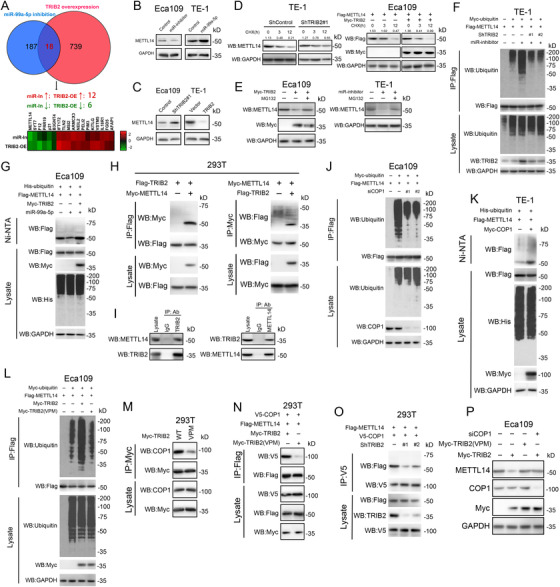
TRIB2 promotes METTL14 proteasomal degradation by recruiting COP1 (A) Venn plot shows the mass spectrum analysis of proteins regulated by miR‐99a‐5p depletion or TRIB2 overexpression in Eca109 cells. Heatmap shows the 18 proteins whose abundance was coincidently co‐regulated by TRIB2 and miR‐99a‐5p. (B) miR‐99a‐5p overexpression upregulated, whereas miR‐99a‐5p inhibition reduced, METTL14 protein levels in ESCC cells, as determined by western blotting. (C) TRIB2 overexpression reduced, whereas TRIB2 inhibition increased, METTL14 protein levels in ESCC cells, as detected by western blotting. (D) Western blotting analysis of METTL14 stability in Eca109 cells transfected with Flag‐METTL14 and Myc‐TRIB2 and of endogenous METTL14 stability in TE‐1 cells expressing TRIB2 shRNA. Upper numbers indicate the intensity of METTL4 relative to GAPDH. (E) Western blotting analysis of METTL14 stability in Eca109 cells transfected with Myc‐TRIB2 (left) or in TE‐1 cells transfected with miR‐99a‐5p inhibitor (right) after treatment with MG132 (100 μM). (F) IP analysis of METTL14 ubiquitination in TE‐1 cells transfected with the indicated constructs, miR‐99a‐5p inhibitor, and/or TRIB2 shRNA. (G) Ni‐nitrilotriacetic acid (Ni‐NTA) pull‐down analysis of METTL14 ubiquitination in Eca109 cells transfected with the indicated constructs and/or with miR‐99a‐5p mimics. (H) Immunoprecipitation (IP) analysis of the interaction between METTL14 and TRIB2 in 293T cells. (I) Reciprocal IP analysis of the interaction between endogenous METTL14 and endogenous TRIB2 in Eca109 cells. (J) IP analysis of METTL14 ubiquitination in Eca109 cells transfected with the indicated constructs and/or with siCOP1. (K) Ni‐NTA pull‐down analysis of METTL14 ubiquitination in TE‐1 cells transfected with the indicated constructs. (L) IP analysis of METTL14 ubiquitination in Eca109 cells transfected with the indicated constructs. (M) IP analysis of the interaction between TRIB2 or its mutant (VPM) and COP1 in 293T cells. (N) IP analysis of the interaction between COP1 and METTL14 in 293T cells transfected with TRIB2 or its mutant (VPM) construct. (O) IP analysis of the interaction between COP1 and METTL14 in 293T cells transfected with the COP1 construct and/or TRIB2 shRNA. (P) Western blotting analysis of METTL14 expression in Eca109 cells transfected with the indicated constructs and/or with siCOP1

We used mass spectrum analysis to identify proteins whose abundance was regulated by TRIB2 overexpression and miR‐99a‐5p inhibition. As a result, the abundance of 739 proteins and 187 proteins were altered by TRIB2 overexpression and miR‐99a‐5p inhibition, respectively. Conspicuously, 18 proteins were coincidently regulated by both miR‐99a‐5p depletion and TRIB2 overexpression (Figure 5A). METTL14, which we found positively regulated miR‐99a‐5p expression, was prominently and consistently suppressed following TRIB2 overexpression and miR‐99a‐5p downregulation (Figure 5B,C). However, the miR‐99a‐5p/TRIB2 axis exhibited no significant effect on the mRNA levels of METTL14 (Figure S6A,B). It appears that METTL14 could decrease TRIB2 expression through upregulating miR‐99a‐5p and is itself suppressed by TRIB2 at the posttranslational level. Consistent with this possibility, CHX chase assays revealed that miR‐99a‐5p overexpression and TRIB2 silencing both stabilized METTL14, while TRIB2 upregulation or miR‐99a‐5p inhibition accelerated METTL14 turnover (Figures 5D and S6C). In addition, proteasome inhibition by MG132 significantly blocked miR‐99a‐5p knockdown‐ or TRIB2 overexpression‐induced proteolysis of METTL14 (Figure 5E).

TRIB2 may serve as an adaptor of the E3 ligase COP1 and inactivate C/EBPα in acute myelogenous leukemia.^14^ We questioned whether TRIB2 also degraded METTL14 in a ubiquitin‐dependent manner. As predicted, METTL14 ubiquitination was greatly enhanced in cells transfected with miR‐99a‐5p inhibitors, compared with control cells and with cells transfected with miR‐99a‐5p inhibitor/TRIB2 shRNA (Figures 5F and S6D). miR‐99a‐5p overexpression suppressed METTL14 ubiquitination, which was fully reversed by TRIB2 overexpression (Figures 5G and S6E). Furthermore, IP assays confirmed that TRIB2 could bind to both endogenous and exogenous METTL14 (Figure 5H,I). TRIB2 is an adaptor protein that possesses a COP1‐binding domain in its C‐terminus (Figure S6F).^15^ Consistent with this finding, COP1 knockdown led to a marked decrease in METTL14 ubiquitination (Figures 5J and S6G). In contrast, COP1 overexpression promoted the ubiquitination of METTL14 in ESCC cells (Figures 5K and S6H). Moreover, TRIB2 harboring amino acid substitutions in its COP1‐binding domain (TRIB2 VPM) exhibited a decreased capacity for COP1‐binding and COP1‐mediated METTL14 ubiquitination, compared with the WT protein (Figures 5L,M and S6I). Furthermore, IP assays revealed that TRIB2 depletion or TRIB2 VPM compromised the ability of COP1 to bind METTL14 (Figures 5N,O and S6J,K). Ultimately, although TRIB2 overexpression in ESCC cells reduced METTL14 abundance, the TRIB2 VPM mutant did not elicit an overt effect, whereas COP1 knockdown fully blocked the TRIB2 overexpression‐induced effect (Figures 5P and S6L).

These results indicate that TRIB2 acts as a substrate adaptor by bridging COP1 to METTL14, inducing the ubiquitination and proteasomal degradation of METTL14. Moreover, we identified a METTL14‐miR‐99a‐5p‐TRIB2 feedback loop in ESCC in which TRIB2 is negatively modulated by METTL14 through miR‐99a‐5p, whereas TRIB2 depletes METTL14 by potentiating METTL14 proteasomal degradation.

### 3.6 TRIB2 activates HDAC2 through the Akt/mTOR/S6K1 signaling pathway in ESCC

**FIGURE 6:**
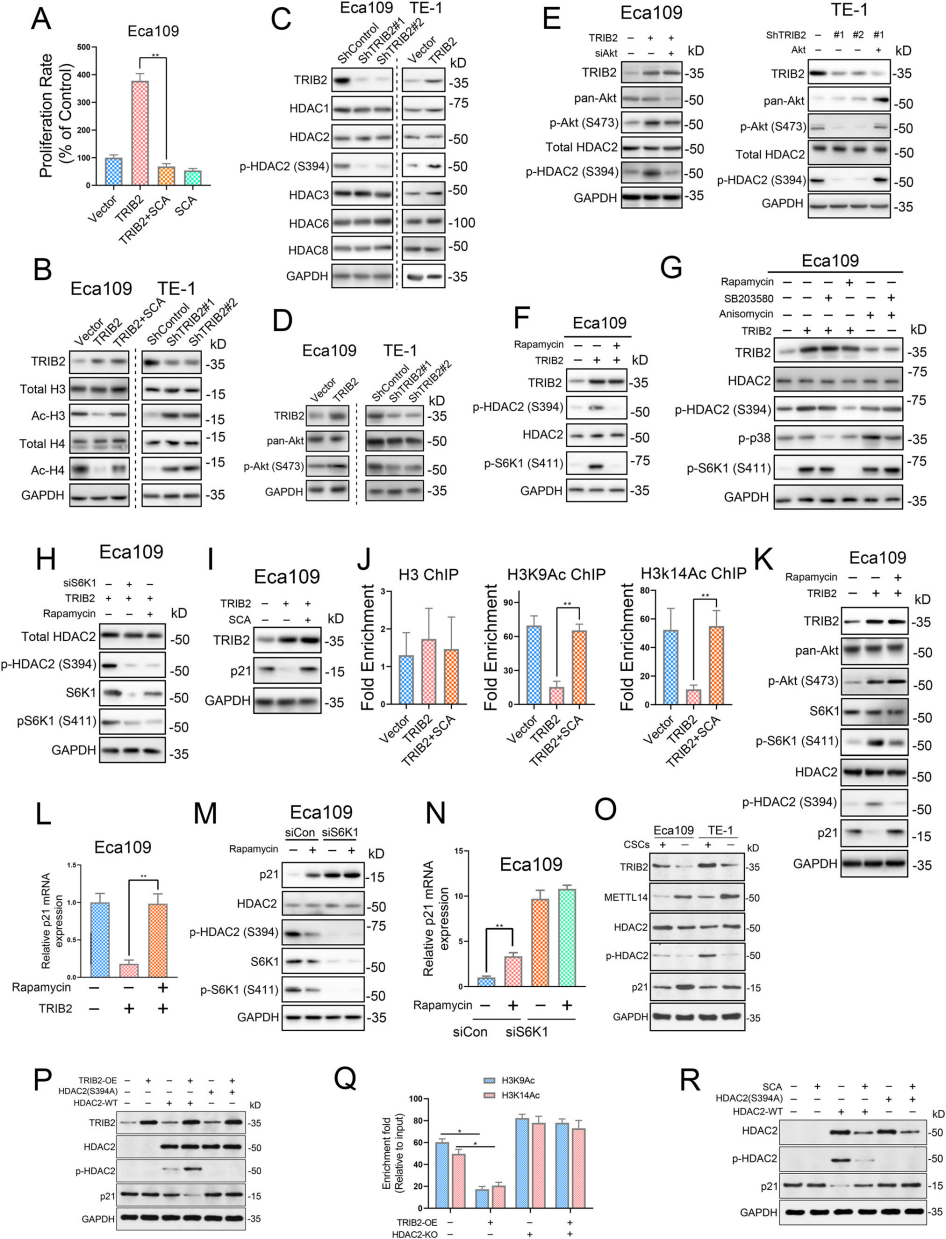
TRIB2‐mediated activation of the Akt/mTOR pathway induces histone deacetylase 2 (HDAC2) phosphorylation and subsequent transcriptional activation of p21 in ESCC cells (A) Cell viability assays showed that HDAC2 inhibition by santacruzamate (SCA; 2 μM) completely blocked TRIB2‐mediated growth of Eca109 cells. (B) The indicated proteins were detected by western blotting of Eca109 and TE‐1 cells after TRIB2 overexpression or silencing. (C) The indicated proteins were detected by western blotting of Eca109 and TE‐1 cells after TRIB2 overexpression or silencing. (D) Phosphorylation of Akt (S473) in ESCC cells after the indicated treatments. (E) The phosphorylation of Akt (S473) and HDAC2 (S394) in ESCC cells transfected with the indicated constructs and/or with siRNA was detected by western blotting. (F) The phosphorylation of HDAC2 (S394) and S6K1 (S411) in Eca109 cells transfected or not with TRIB2 constructs was detected by western blotting after treatment of the cells with rapamycin (100 nM). (G) The phosphorylation of S6K1, p38, and HDAC2 in Eca109 cells transfected with the indicated constructs were detected by western blotting after treatment of the cells with rapamycin (100 nM), SB203580 (10 μM), or anisomycin (1 g/ml). (H) The phosphorylation of S6K1 (S411) and HDAC2 (S394) in Eca109 cells transfected with TRIB2 constructs and/or with S6K1 siRNA was detected by western blotting after treatment of the cells with or without rapamycin (100 nM). (I) The protein expression of p21 in Eca109 cells transfected or not with TRIB2 constructs was detected by western blotting after treatment of the cells with an HDAC2 inhibitor. (J) ChIP‐PCR was performed using the indicated ESCC cells to detect H3K9Ac and H3K14Ac enrichment at p21 promoter regions after treatment of the cells with or without an HDAC2 inhibitor (SCA, 2 μM). (K) The protein levels of p21 and phosphorylation of Akt (S473), S6K1 (S411), and HDAC2 (S394) in Eca109 cells transfected with or without TRIB2 constructs were detected by western blotting after the treatment of the cells with rapamycin (100 nM). (L) The mRNA levels of p21 in Eca109 cells transfected with or without TRIB2 constructs were detected by RT‐PCR after treatment of the cells with rapamycin (100 nM). (M) The protein levels of p21 and phosphorylation of S6K1 (S411) and HDAC2 (S394) in Eca109 cells transfected or not with S6K1 siRNA were detected by western blotting after treatment with rapamycin (100 nM). (N) The mRNA levels of p21 in Eca109 cells transfected or not with S6K1 siRNA were detected by RT‐PCR after treatment of the cells with rapamycin (100 nM). (O) The expression of METTL14, TRIB2, p21, HDAC2, and its phosphorylated form in CSC and non‐CSC subpopulations of ESCC cells were analyzed by western blotting. (P) The abundance of total‐HDAC2, p‐HDAC2, and p21 in the HDAC2‐KO or WT 293T cells transfected with the indicated constructs were determined by western blotting. (Q) ChIP‐PCR was performed in the HDAC2‐KO or WT 293T cells with or without TRIB2 overexpression to detect H3K9Ac and H3K14Ac enrichment at p21 promoter regions. (R) The abundance of total‐HDAC2, p‐HDAC2, and p21 in the HDAC2‐KO or WT 293T cells with or without SCA treatment was determined by western blotting. The data are presented as the mean ± SD. *p < 0.05, **p < 0.01, ***p < 0.001. *p‐*values were determined by one‐way ANOVA with Tukey's post hoc test

Intriguingly, although treatment with HDAC inhibitors had no overt effect on miR‐99a‐5p expression (Figure S3E), we observed that HDAC2 inhibitors (SCA, 2 μM) largely blocked TRIB2‐mediated growth of ESCC cells (Figures 6A and S7A). This finding suggested a potential role of TRIB2 in regulating epigenetic processes, possibly through activation of HDAC2. Consistent with this possibility, TRIB2 knockdown resulted in increased ac‐H3 and ac‐H4 levels, while TRIB2 overexpression dramatically decreased ac‐H3/H4, and HDAC2 inhibition largely rescued this effect (Figure 6B). Assessment of the protein expression of HDACs revealed that HDAC2 phosphorylation at Ser394 was regulated by TRIB2 (Figure 6C).

Akt activity is tightly modulated by TRIB2,^16^ we also confirmed that TRIB2 was able to induce Akt phosphorylation in ESCC (Figure 6D). This finding led us to hypothesize that the mechanism by which TRIB2 activates HDAC2 is mediated through the Akt pathway. Consistently, Akt depletion decreased TRIB2‐induced phosphorylation of HDAC2 (Ser394), whereas decreased HDAC2 phosphorylation was reversed by Akt overexpression (Figure 6E). Moreover, TRIB2‐induced phosphorylation of HDAC2 was strongly blocked by rapamycin, an mTOR inhibitor (Figure 6F). In addition, although treatment with anisomycin, an activator of both p38 and p70S6K1, induced HDAC2 phosphorylation in Eca109, rapamycin but not SB203580 (an inhibitor of p38) abrogated TRIB2 overexpression‐mediated HDAC2 phosphorylation (Figure 6G). These data suggest that the Akt/mTOR/p70S6K1 pathway might mediate TRIB2‐dependent HDAC2 phosphorylation.

We further silenced p70S6K1 and found that TRIB2 no longer induced HDAC2 phosphorylation under these conditions (Figure 6H). p21 is closely involved in the attenuation of cancer cell stemness and the enhancement of the radiotherapy response, and TRIB2 can regulate p21 via interaction with AP4 in colorectal cancer.^17^, ^18^ Our mass spectrum results also confirmed that TRIB2 overexpression or miR‐99a‐5p inhibition decreased p21 abundance (Figure 5A). However, although TRIB2 decreased p21 expression in ESCC cells, this effect was almost completely blocked after treatment with SCA (Figure 6I). This observation indicates that TRIB2 regulates p21 expression independently of AP4 and that HDAC2‐mediated deacetylase activity may be necessary for p21 suppression in ESCC. Consistently, the promoter of p21 exhibited decreased H3K9Ac or H3K14Ac in TRIB2‐overexpressing ESCC cells, which was largely reversed by SCA (Figure 6J). Immunoblotting and PCR showed that mTOR inhibition significantly elevated p21 levels and that S6K1 was essential for the effect of rapamycin on p21 expression (Figure 6K–N). Western blotting analysis of CSC and non‐CSC populations of ESCC cells further confirmed that TRIB2 and phosphorylated HDAC2 (S394) levels were significantly elevated, while METTL14 and p21 levels were markedly decreased in CSCs of ESCC cells (Figure 6O). To further confirm whether the phosphorylation of HDAC2 at S394 determines TRIB2‐mediated p21 deacetylation, we constructed an HDAC2 S394 mutant construct (S394A) expressing a mutant HDAC2 with an alanine‐to‐serine substitution at position 394 and re‐expressed HDAC2 or the S394A mutant in HDAC2 knockout HEK293T cells with or without TRIB2 overexpression. We found that HDAC2 deletion in 293T cells blocked the suppressive effect of TRIB2 on p21 expression, which was rescued by HDAC2‐WT but not by a phosphor‐deficient HDAC2 mutant (Figure 6P). TRIB2 overexpression‐induced deacetylation on the p21 promoter was fully abrogated in 293T cells with HDAC2 knockout (Figure 6Q). Moreover, SCA failed to downregulate p21 expression in the HDAC2 KO cells or the cells with HDAC2‐S394 re‐expression, compared with the cells re‐expressing WT HDAC2 (Figure 6R).

These data suggested that p70S6K1‐mediated phosphorylation of HDAC2 (Ser394) following TRIB2‐induced Akt/mTOR activation is essential for the epigenetic suppression of p21 and that it might promote CSC characteristics and radioresistance in ESCC.

### 3.7 Disruption of Akt/mTOR/S6K1 axis‐mediated HDAC2 (Ser394) phosphorylation inhibits cancer stemness and ameliorates the radioresistance of ESCC cells

**FIGURE 7:**
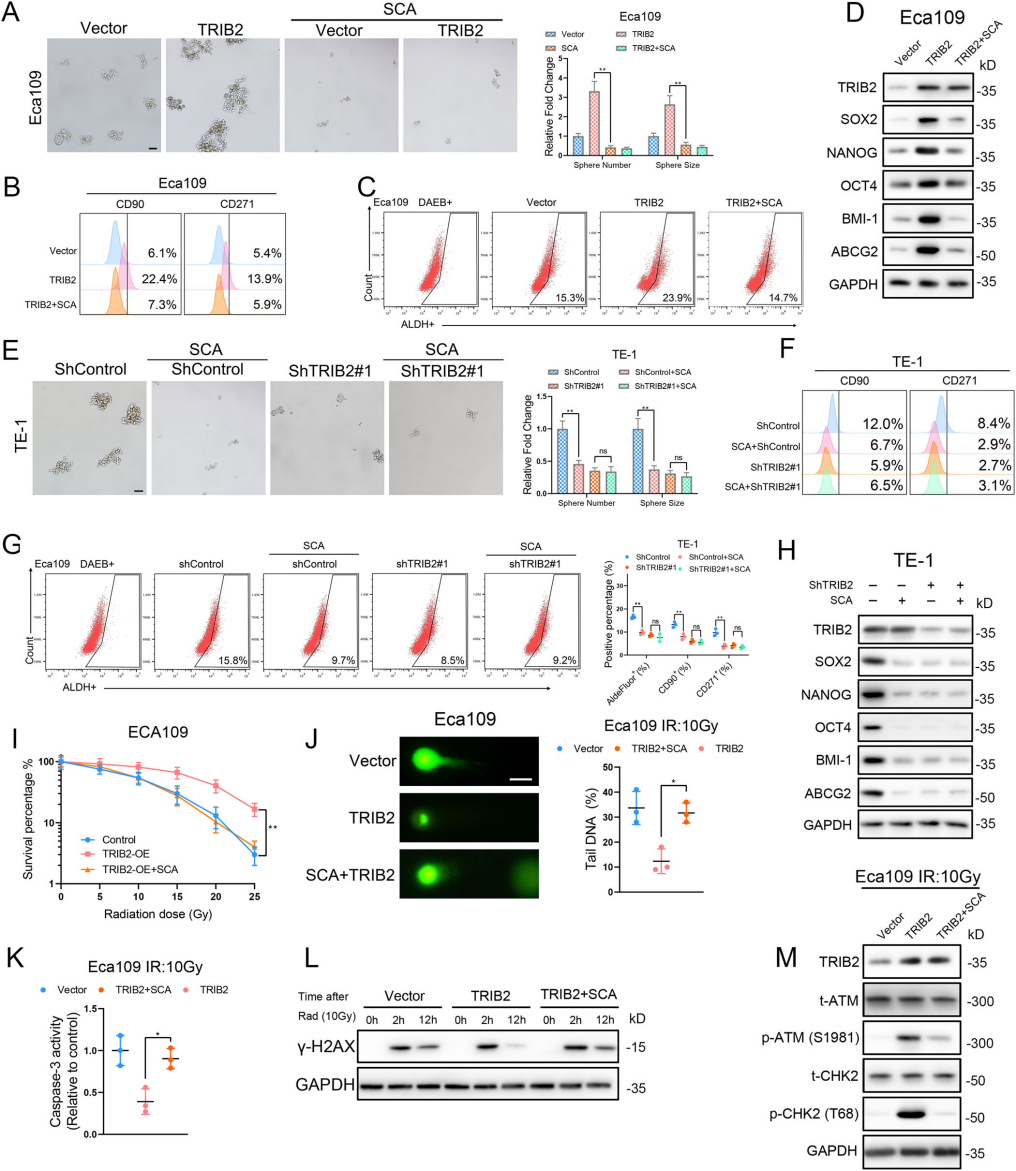
HDAC2 is essential for the TRIB2‐mediated CSC properties and radioresistance of ESCC cells (A) Representative images (left) and statistical quantification (right) of sphere formation in the indicated ESCC cells treated with or without the HDAC2 inhibitor (SCA, 2 μM). Scale bars: 100 μm. (B–C) Representative images and statistical quantification of the flow cytometric analysis determining the ALDH activity and proportion of the CD90‐positive and CD271‐positive subpopulations in the indicated ESCC cells treated with or without HDAC2 inhibitor (SCA, 2 μM). (D) Western blotting analysis of CSC markers in the indicated ESCC cells treated with or without the HDAC2 inhibitor (SCA, 2 μM). (E) Representative images (left) and statistical quantification (right) of sphere formation by the indicated ESCC cells treated with or without the HDAC2 inhibitor (SCA, 2 μM). Scale bars: 100 μm. (F‐G) Representative images and statistical quantification of the results of flow cytometric analyses to determine ALDH activity and the relative proportions of CD90^+^ and CD271^+^ cells in the indicated ESCC cells treated with or without HDAC2 inhibitor (SCA, 2 μM). (H) Western blotting analysis of CSC markers in the indicated Eca109 cells treated with or without an HDAC2 inhibitor (SCA, 2 μM). (I) Clonogenic assay of the indicated ESCC cells with or without an HDAC2 inhibitor (SCA, 2 μM) at an IR dose of 10 Gy. The survival fraction was calculated as (number of colonies formed/number of cells plated)_irradiated_/(number of colonies formed/number of cells plated)_control_. (J) Relative caspase‐3 activity in the indicated ESCC cells 24 h after an IR dose of 10 Gy. (K) Representative images (left) and statistical quantification (right) of the comet assay results in the indicated ESCC cells 6 h after an IR dose of 10 Gy. Scale bars: 10 μm. (L) After treatment with 10‐Gy IR, the indicated ESCC cells were cultured under normal conditions for 2 and 12 h, and γ‐H2AX expression in each group was detected by western blotting. (M) Western blotting analysis of the total and phosphorylated proportion of checkpoint markers ATM and CHK in the indicated ESCC cells 4 h after an IR dose of 10 Gy. The data are presented as the mean ± SD. *p < 0.05, **p < 0.01, ***p < 0.001. *p‐*values were determined by one‐way ANOVA with Tukey's post hoc test

We further investigated the involvement of HDAC2 in TRIB2‐driven cancer stemness and radioresistance. The functional study revealed that inhibition of HDAC2 strongly weakened the effects of TRIB2 on the sphere‐formation capacity, CSC marker expression, and CD90^+^ and CD271^+^ subpopulations of ESCC cells (Figure 7A–D). Moreover, TRIB2‐knockdown TE‐1 cells were not sensitive to SCA treatment (Figure 7E–H). In addition, although SCA suppressed the growth of ESCC CSCs, it had little effect on non‐stem ESCC cells (Figure S7B,C). These results suggest that HDAC2 inhibition is the direct target of TRIB2 and that such inhibition disrupts ESCC CSC growth and maintenance.

The assessment of DNA repair efficiency demonstrated that TRIB2‐enhanced DNA repair after IR was dramatically blocked by HDAC2 inhibition, as indicated by colony‐formation and apoptosis assays, DNA repair checkpoint response, comet assays, and γ‐H2AX expression (Figure 7I–M). Ultimately, SCA treatment significantly sensitized TE‐1 cells to radiotherapy, and HDAC2 inhibition had a greater effect on cells transfected with shControl than on cells treated with shTRIB2, based on the results of colony formation, apoptosis, and DNA damage response assays (Figure S7D–H). These findings indicate that activation of HDAC2 is critical for TRIB2‐promoted radioresistance and cancer stemness of ESCC cells.

### 3.8 Disruption of the TRIB2/HDAC2 axis sensitizes ESCC PDXs to radiotherapy

**FIGURE 8:**
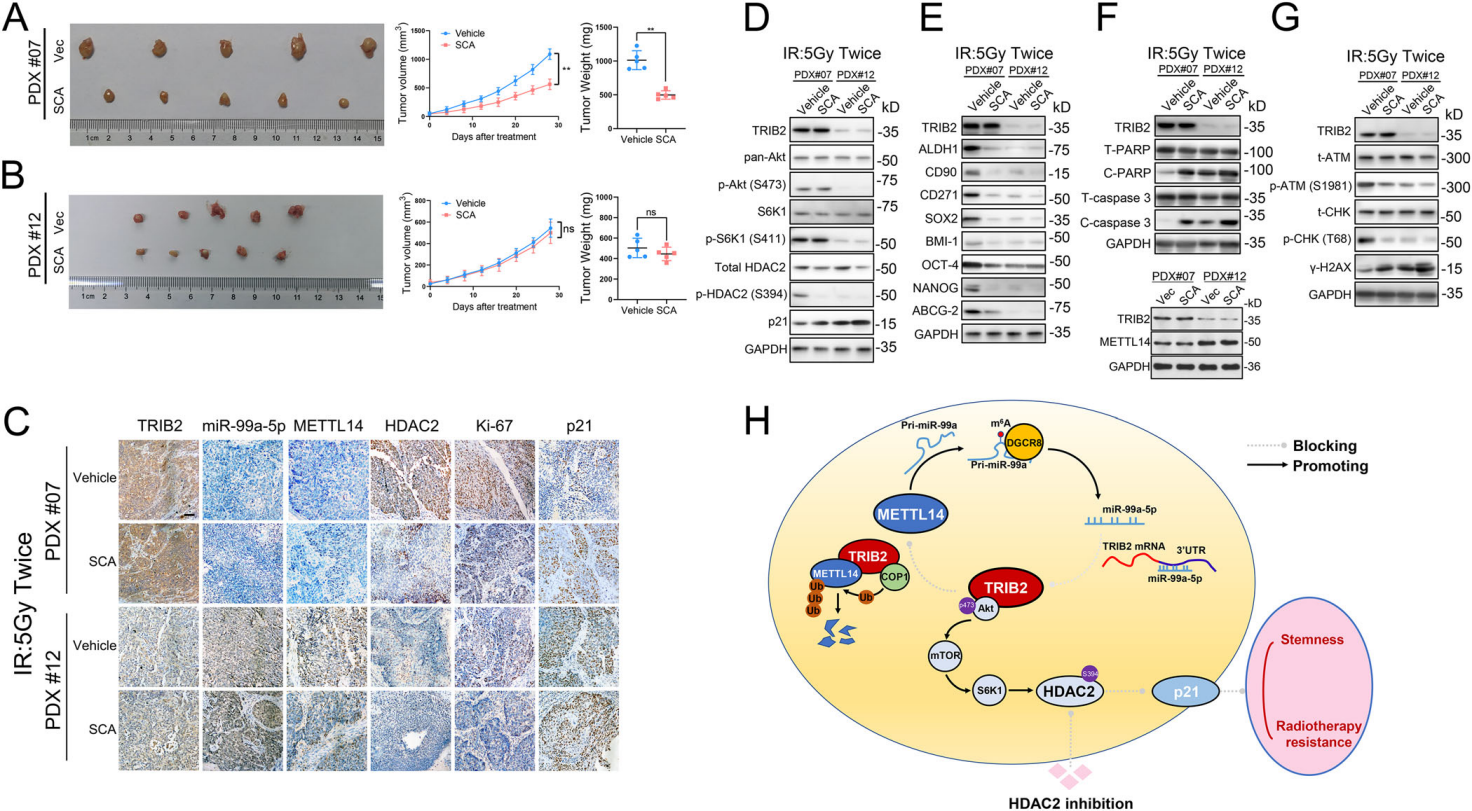
SCA administration suppressed tumor growth in ESCC PDXs (A‐B) ESCC‐derived xenografts expressing TRIB2 at different levels were treated with vehicle, SCA (50 mg/kg/day, intraperitoneal injection), and IR (5 Gy, twice). A tumor volume growth curve and the tumor weights are presented. (C) ISH of miR‐99a‐5p abundance and IHC of the indicated proteins in the patient‐derived xenografts (PDXs) of the indicated groups. (D‐G) Western blotting analyses of the indicated markers in PDXs of the indicated groups. (H) Schematic diagram describing the METTL14/miR‐99a‐5p/TRIB2 positive feedback circuit and molecular mechanism of TRIB2‐mediated radioresistance and CSC characteristics of ESCC. The data represent the mean ± SD. Scale bars: 50 μm. *p < 0.05, **p < 0.01, ***p < 0.001. *p‐*values were determined by the unpaired two‐tailed Student's *t*‐test

We next investigated the role of TRIB2/HDAC2 in a PDX model. PDX tumor #07, which has high TRIB2 expression, and PDX tumor #12, which has low TRIB2 expression, were used in these experiments. SCA administration markedly decreased the growth and weight of PDX tumors with high TRIB2 expression following IR. In contrast, the low TRIB2‐expressing PDX tumor was not sensitive to SCA treatment following IR (Figure 8A,B). IHC staining and western blotting of xenografted tumors further confirmed that the METTL14/miR‐99a‐5p/TRIB2 feedback loop, as well as the Akt/mTOR/S6K1/HDAC2 axis, were involved in this response (Figure 8C–F) as demonstrated in vitro. Moreover, the CSC properties were significantly reduced, whereas p21 expression was greatly enhanced in TRIB2‐high but not in TRIB2‐low tumor xenografts following SCA treatment (Figure 8C–E). HDAC2 inhibition inhibited the proliferation of ESCC cells in the TRIB2‐high PDX group but failed to influence tumor growth in the TRIB2‐low PDX group as determined by IHC staining of Ki‐67, indicating the biological relevance of the TRIB2/HDAC2 axis in attenuating radiotherapy‐mediated growth suppression in ESCC (Figure 8C). Cleaved caspase‐3 and γ‐H2AX expression were substantially increased, and the DNA damage checkpoint response was significantly suppressed, following SCA administration in TRIB2‐high PDX tumors, compared with TRIB2‐low PDX tumors treated with IR (Figure 8F,G).

## 4 DISCUSSION

The present unfavorable outcome of ESCC indicates that a more thorough and comprehensive understanding of the molecular mechanism underlying ESCC progression and radioresistance is urgently needed. Our work revealed that the METTL14/miR‐99a‐5p/TRIB2 feedback circuit is a positive regulator in maintaining CSC characteristics and enhancing the radioresistance of ESCC. Our data also demonstrated that TRIB2 activates HDAC2 via the Akt signaling pathway. Activation of HDAC2 was found to epigenetically repress p21 expression, resulting in enhanced radioresistance and cancer stemness of ESCC cells. This work describes a new feedback circuit/regulatory mechanism in ESCC and will aid in the further understanding of ESCC progression and management.

Radiotherapy failure is common among patients with advanced ESCC and is associated with CSC persistence.^19^ However, studies on the initiation and maintenance of cancer cell stemness in ESCC are limited. Although miR‐99a‐5p has been reported to suppress the proliferation and metastasis of ESCC cells,^20^ this study is the first to find that downregulated miR‐99a‐5p in ESCC is essential for CSC properties and the radioresistance of ESCC cells. Consistent with these results, miR‐99a‐5p has been characterized as a tumor suppressor in oral carcinoma,^21^ head and neck squamous cell carcinoma,^22^ breast cancer,^23^ and lung adenocarcinoma.^24^ Thus, miR‐99a‐5p could act through multiple mechanisms to suppress tumor cell aggressiveness. Clinically, we showed that miR‐99a‐5p was closely linked with favorable response in patients undergoing radiotherapy (Figure 2L–N), making it a potential valuable marker for the early prediction and stratification of patients based on the benefits gained from radiotherapy.

Although TRIB2 is associated with aggressiveness of malignancies,^25^, ^26^ little is known regarding its exact role in ESCC. We demonstrated that miR‐99a‐5p could inhibit TRIB2 expression by targeting its 3′ UTR and downregulation of miR‐99a‐5p accounted for TRIB2 hyperactivation and, subsequently, the promotion of CSC properties and radioresistance of ESCC cells. It has been acknowledged that CSCs are mainly responsible for the development of radioresistance and tumor relapse in ESCC.^19^ However, in the clinical practice of ESCC treatment, drugs targeting CSCs are still lacking. The identification of TRIB2 as a key player participating in CSC properties and radioresistance development in ESCC makes it a value pharmaceutical target. Therapeutically targeting TRIB2 in ESCC also has other potential advantages: (1) TRIB2 was critical for the maintenance of CSC properties and their renewal ability, thus TRIB2 disruption would eradicate CSCs and restore radiosensitivity. (2) TRIB2 was preferably upregulated in CSC subpopulations of ESCC cells, compared with non‐tumorous cells. Its impact on cell growth is CSC‐specific; the inhibition of TRIB2 in normal esophageal epithelial cells exhibited little cytotoxicity (data not shown), indicating that pharmaceutical disruption of TRIB2 would have a favorable therapeutic index. (3) TRIB2 is a kinase‐like protein with ATP‐binding ability, making it an amenable target for specific and reversible inhibitors.^27^ Collectively, the development of highly effective and specific TRIB2‐targeting inhibitors will be explored in future studies. m^6^A modification plays pivotal roles in miRNA processing and splicing.^28^, ^29^ METTL14 has been shown to act as an antitumor effector through its N^6^‐methyladenosine‐modifying function.^30^, ^31^, ^32^ Consistently, we demonstrated that METTL14 downregulation in ESCC abrogated the inhibitory effect of miR‐99a‐5p on TRIB2 expression by hindering the biogenesis of mature miR‐99a‐5p from primary mir‐99a and that this, in turn, increased the CSC characteristics and radioresistance of ESCC. Although TRIB2 can bind to the RING ubiquitin ligase E3 COP1,^15^ we showed for the first time that METTL14 is a binding partner of TRIB2 and also a substrate of COP1. Specifically, TRIB2 binds to METTL14 and thereby serves as a substrate adaptor that facilitates COP1‐mediated proteasomal degradation of METTL14. These regulatory mechanisms involving METTL14/miR‐99a‐5p/TRIB2 create a positive feedback circuit in ESCC. TRIB2 overexpression leads to increased METTL14 inhibition via COP1‐mediated proteasomal degradation, and suppression of m^6^A activity due to METTL14 degradation results in processing defects and decreased biogenesis of mature miR‐99a‐5p from primary miR‐99a. When miR‐99a‐5p was expressed at low levels, its ability to sponge TRIB2 mRNA was abolished, and TRIB2 expression increased further due to the reduced inhibition by miR‐99a‐5p/METTL14. To our knowledge, this study is the first to unveil this feedback loop and the underlying regulatory mechanisms in ESCC.

Transcription efficiency is balanced between activation by histone acetyltransferases and inhibition by HDACs.^33^ Richard et al. reported that TRIB2 promotes the phosphorylation of Akt at Ser471 via its COP1 domain.^16^ Consistent with this finding, we showed that TRIB2‐induced Akt activation was relayed from mTOR to S6K1 and eventually led to the phosphorylation of HDAC2. Although TRIB2 has been reported to modulate p38,^34^ p38 inhibition did not abrogate the anisomycin‐induced phosphorylation of HDAC2 in ESCC cells, suggesting that the effect of TRIB2 on the MAPK pathway might be cancer‐dependent. It is clear that the repression of p21 in malignancies tightly correlates with CSC properties and radioresistance^35^, ^36^ and that TRIB2 inhibits p21 via AP4 in colorectal cancer.^18^ Here, we unveiled a novel regulatory mechanism in ESCC in which TRIB2‐induced phosphorylation and activation of HDAC2 led to the deacetylation and transcriptional repression of p21, and the TRIB2/HDAC2 axis might be the dominant mechanism responsible for p21 suppression in ESCC, which ultimately mediates the effect of TRIB2 on CSC properties and radioresistance in ESCC.

It is particularly noteworthy that a PDX model using human clinical ESCC specimens substantiates our in vitro results showing that TRIB2 strongly induced radioresistance, while HDAC2 inhibition effectively blocked the effects of TRIB2 and strengthened the suppression of tumor growth by radiotherapy. This result strongly supports the clinical data and in vitro function of TRIB2 in mediating radioresistance in ESCC.

## 5 CONCLUSION

In summary, we identified a key regulatory METTL14‐miR‐99a‐5p‐TRIB2 feedback circuit that epigenetically represses p21 through Akt/mTOR/S6K1/HDAC2 to promote cancer stemness and radioresistance in ESCC (Figure 8H). These components provide promising effective targets for antitumor therapies in ESCC patients.

## AUTHOR CONTRIBUTIONS

Zhenchuan Liu and Yongxin Zhou designed the study. Data analysis, figure preparation, manuscript drafting, and most of the experiments were performed by Zhenchuan Liu. Wenli Wang, Shiliang Xie, Shaorui Gu, Kaiqing Wu, Xishi Wang, Tiancheng Lu, Lei Li, and Chenglai Dong assisted with some of the experiments. All authors read and approved the final manuscript.

## ETHICS STATEMENT

This study was approved by the Medical Ethics Committee of Shanghai Tongji Hospital. Informed consent was obtained from each patient before enrollment in this study. All animal experiments were approved by the Animal Care and Use Committee of Shanghai Tongji Hospital and conducted in accordance with ethical standards.

## CONFLICT OF INTEREST

The authors declare that they have no competing interests.

## Supporting information

SUPPORTING INFORMATION

SUPPORTING INFORMATION

SUPPORTING INFORMATION

SUPPORTING INFORMATION

SUPPORTING INFORMATION

SUPPORTING INFORMATION

SUPPORTING INFORMATION

SUPPORTING INFORMATION

SUPPORTING INFORMATION

SUPPORTING INFORMATION

SUPPORTING INFORMATION

SUPPORTING INFORMATION

## Data Availability

All data from this study can be obtained from the corresponding author upon reasonable request.
