# CORRIGENDUM

**DOI:** 10.1002/ctm2.741

**Published:** 2022-06-27

In the article by Yongxin Zhou et al., (2021), the authors noted some minor errors in Figure 7A and Figure 8C. Specifically: In Figure 7A, the image of ECA109 with SCA treatment was misused from TE1 cells in Figure 7E. In Figure 8C, an incorrect image was shown for the miR‐99a‐5p staining in PDX#07 plus SCA administration. The authors apologize for any inconvenience caused by this error. The corrected figures are given below. The article has been corrected online.

**FIGURE 7:**
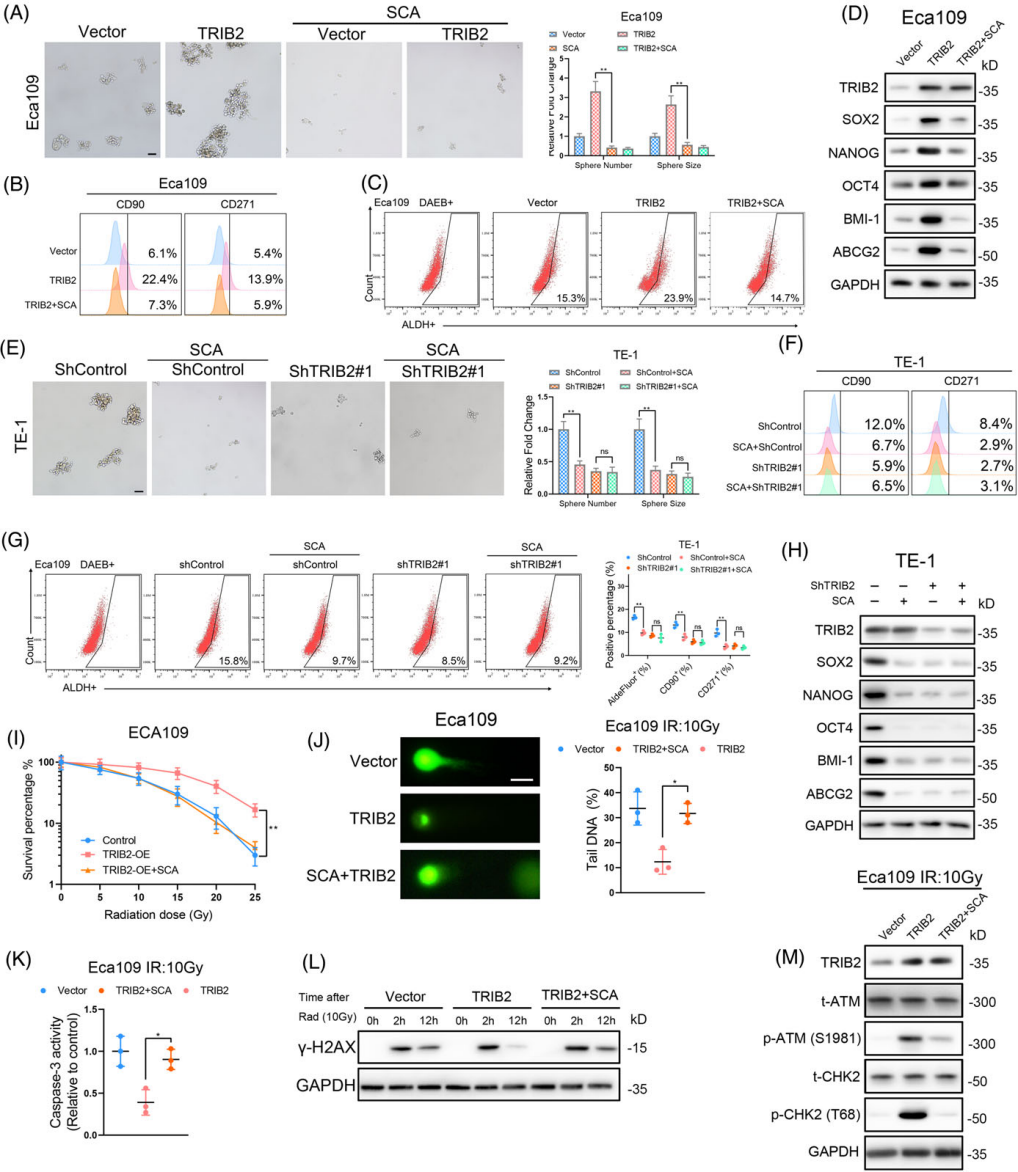
HDAC2 is essential for the TRIB2‐mediated CSC properties and radio resistance of ESCC cells (A) Representative images(left) and statistical quantification (right) of sphere formation in the indicated ESCC cells treated with or without the HDAC2 inhibitor (SCA,2µM). Scale bars: 100µm. (B–C) Representative images and statistical quantification of the flow cytometric analysis determining the ALDH activity and proportion of the CD90‐positive and CD271‐positive subpopulations in the indicated ESCC cells treated with or without HDAC2inhibitor (SCA, 2µM). (D) Western blotting analysis of CSC markers in the indicated ESCC cells treated with or without the HDAC2 inhibitor(SCA, 2µM). (E) Representative images (left) and statistical quantification (right) of sphere formation by the indicated ESCC cells treated with or without the HDAC2 inhibitor (SCA, 2µM). Scale bars: 100µm. (F‐G) Representative images and statistical quantification of the results of flow cytometric analyses to determine ALDH activity and the relative proportions of CD90+and CD271+cells in the indicated ESCC

**FIGURE 8:**
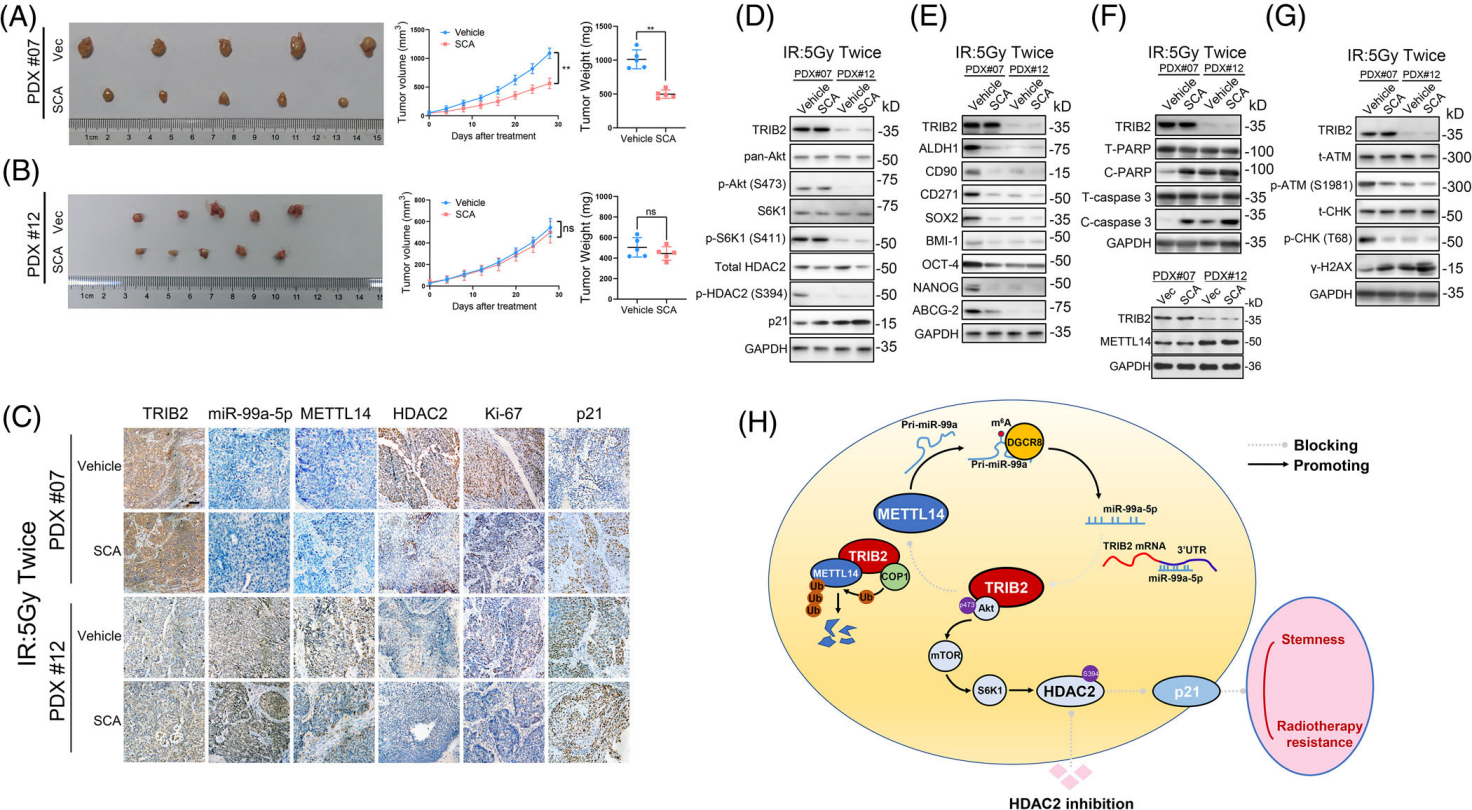
SCA administration suppressed tumor growth in ESCC PDXs (A‐B) ESCC‐derived xenografts expressing TRIB2 at different levels were treated with vehicle, SCA (50 mg/kg/day, intraperitoneal injection), and IR (5 Gy, twice). A tumor volume growth curve and the tumor weights are presented. (C) ISH of miR‐99a‐5p abundance and IHC of the indicated proteins in the patient‐derived xenografts (PDXs) of the indicated groups. (D‐G) Western blotting analyses of the indicated markers in PDXs of the indicated groups. (H) Schematic diagram describing the METTL14/miR‐99a‐5p/TRIB2 positive feedback circuit and molecular mechanism of TRIB2‐mediated radio resistance and CSC characteristics of ESCC. The data represent the mean±SD. Scale bars: 50µm. *p<0.05, **p<0.01, ***p<0.001.p‐values were determined by the unpaired two‐tailed Student's t‐test

These corrections have no impact on the experimental outcome or conclusions.
