# Supplementary figures and images for: A methyltransferase‐like 14/miR‐99a‐5p/tribble 2 positive feedback circuit promotes cancer stem cell persistence and radioresistance via histone deacetylase 2‐mediated epigenetic modulation in esophageal squamous cell carcinoma
Source: Clin Transl Med. 2021 Sep 15;11(9):e545. doi: 10.1002/ctm2.545 (PMC8441142; doi:10.1002/ctm2.545)

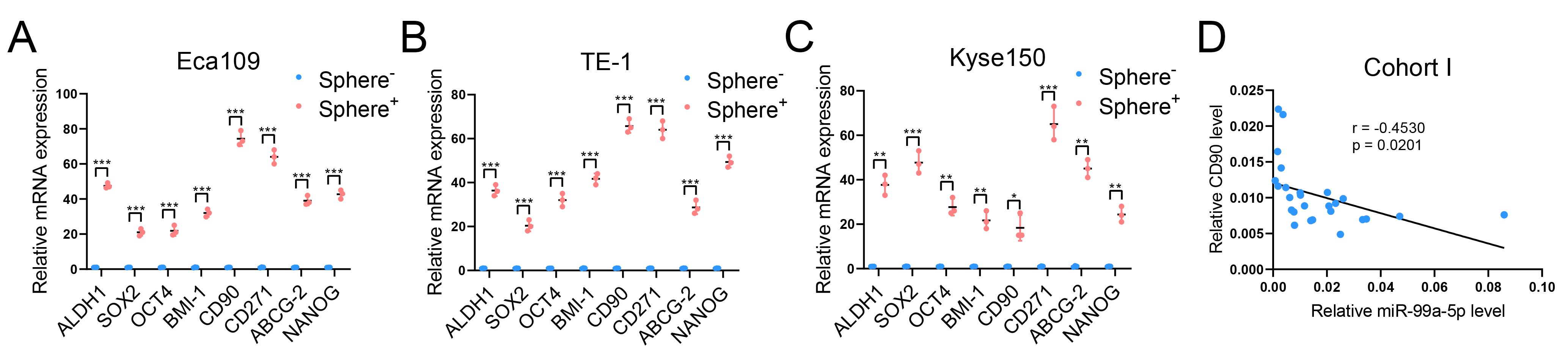

Supplement: Supplementary file 1 — SUPPORTING INFORMATION [file CTM2-11-e545-s002.tif]

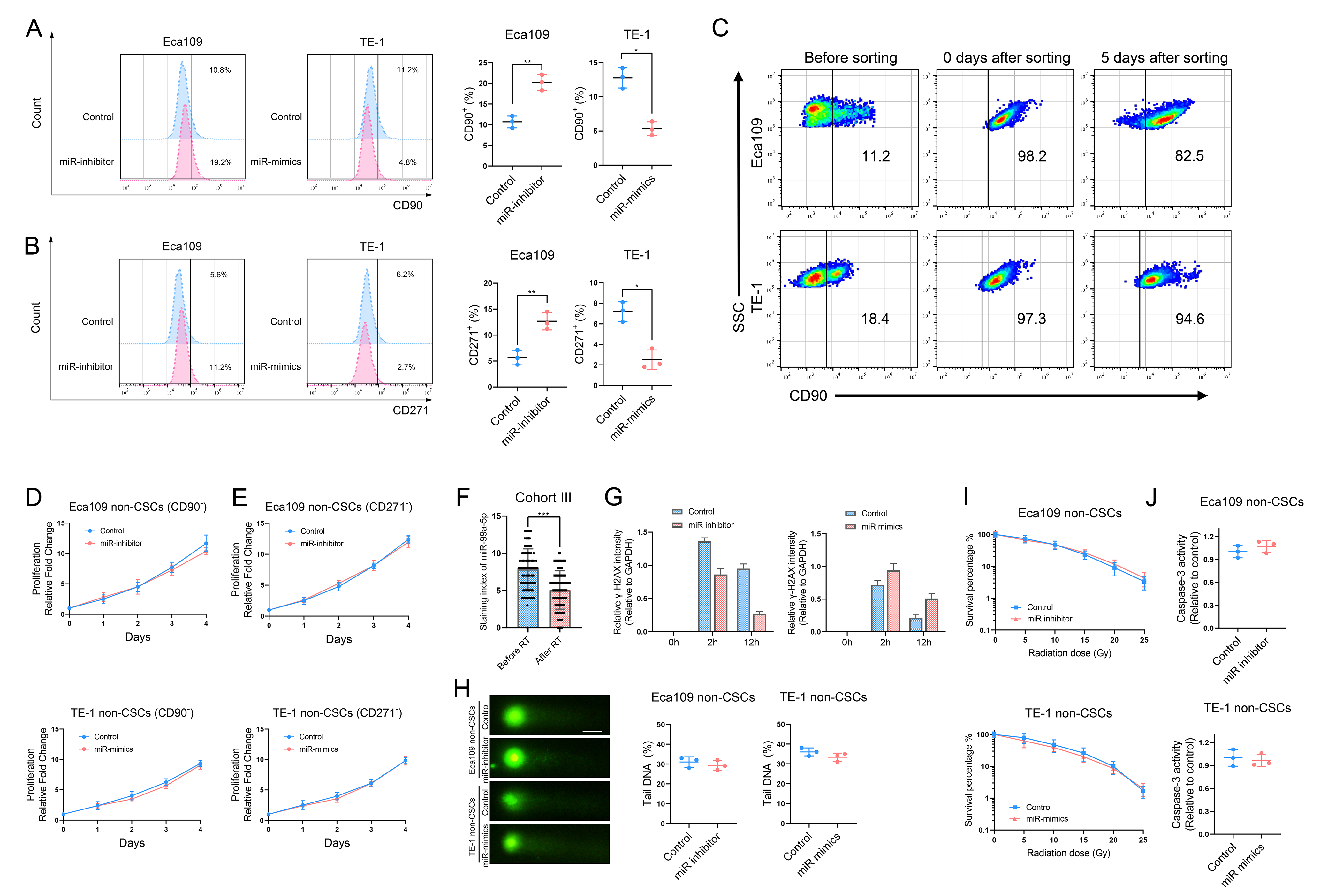

Supplement: Supplementary file 2 — SUPPORTING INFORMATION [file CTM2-11-e545-s012.tif]

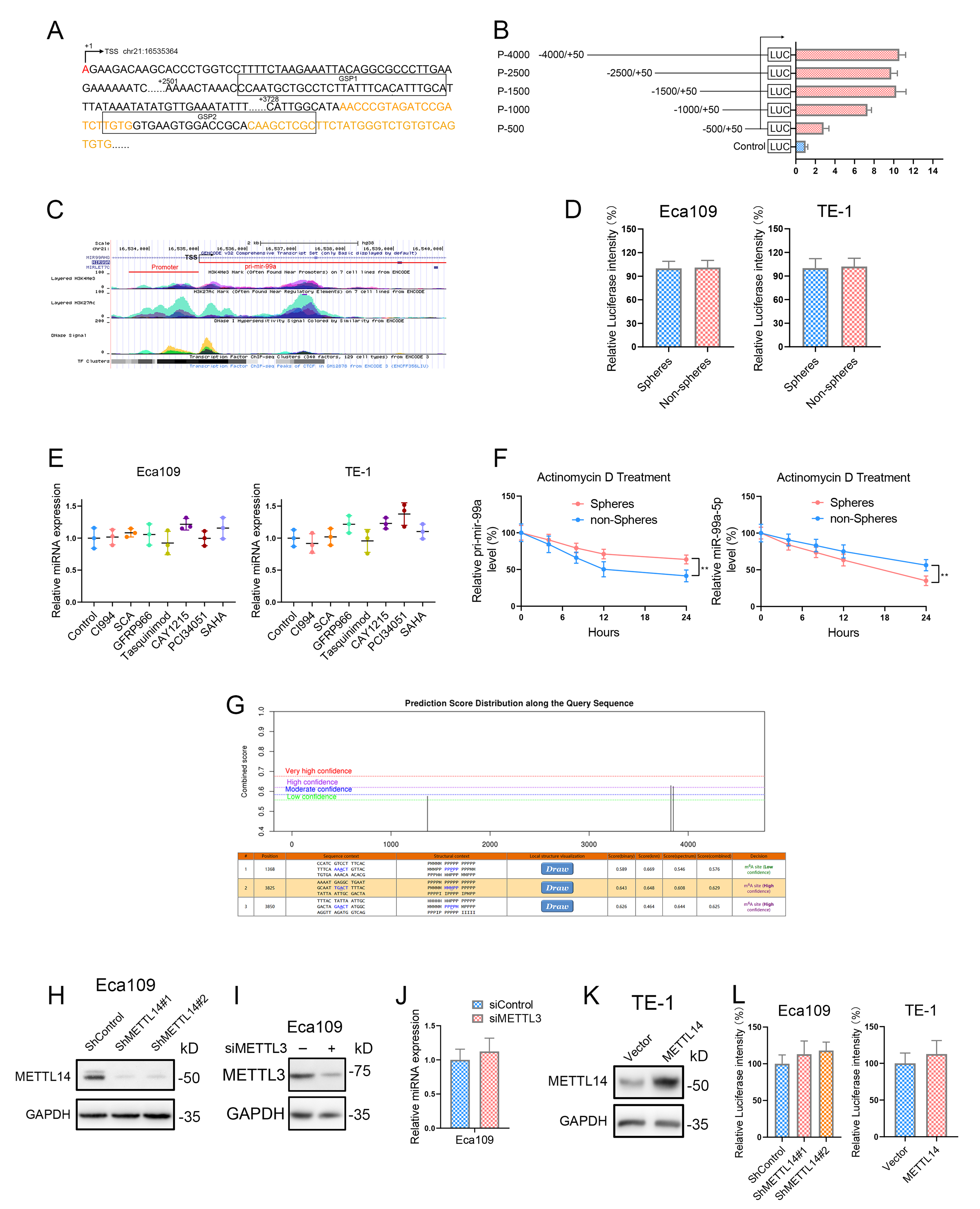

Supplement: Supplementary file 3 — SUPPORTING INFORMATION [file CTM2-11-e545-s010.tif]

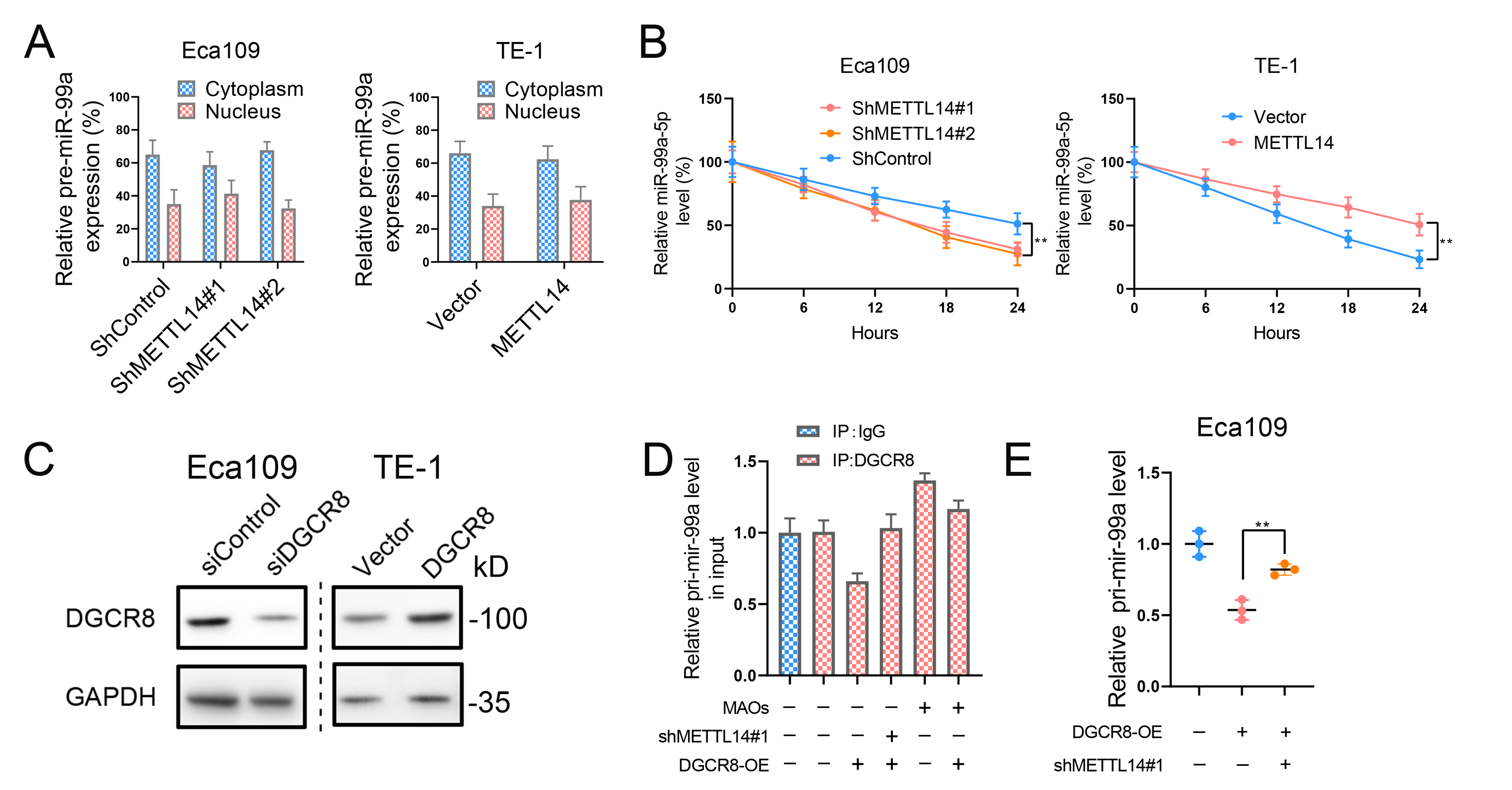

Supplement: Supplementary file 4 — SUPPORTING INFORMATION [file CTM2-11-e545-s006.tif]

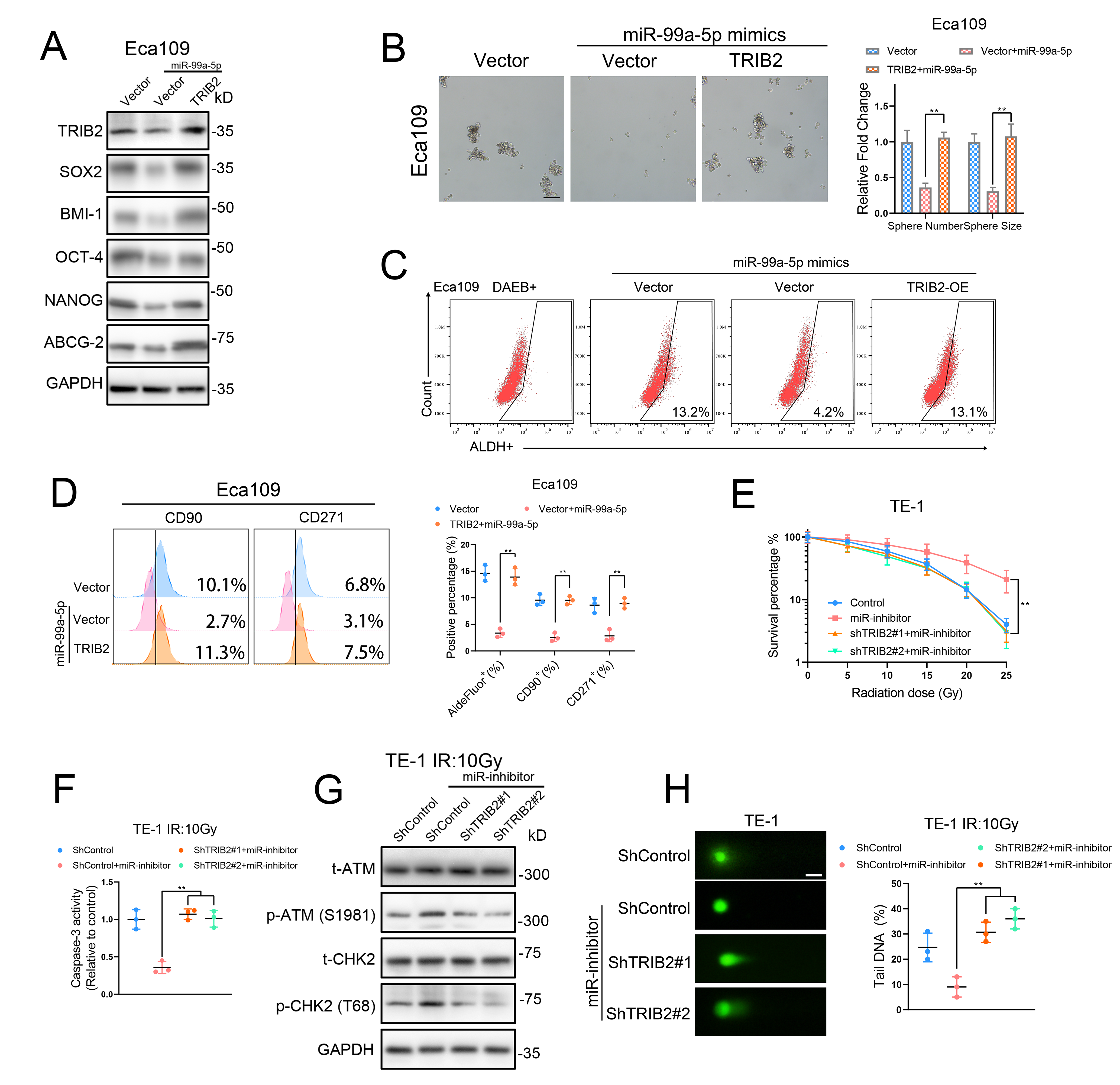

Supplement: Supplementary file 5 — SUPPORTING INFORMATION [file CTM2-11-e545-s003.tif]

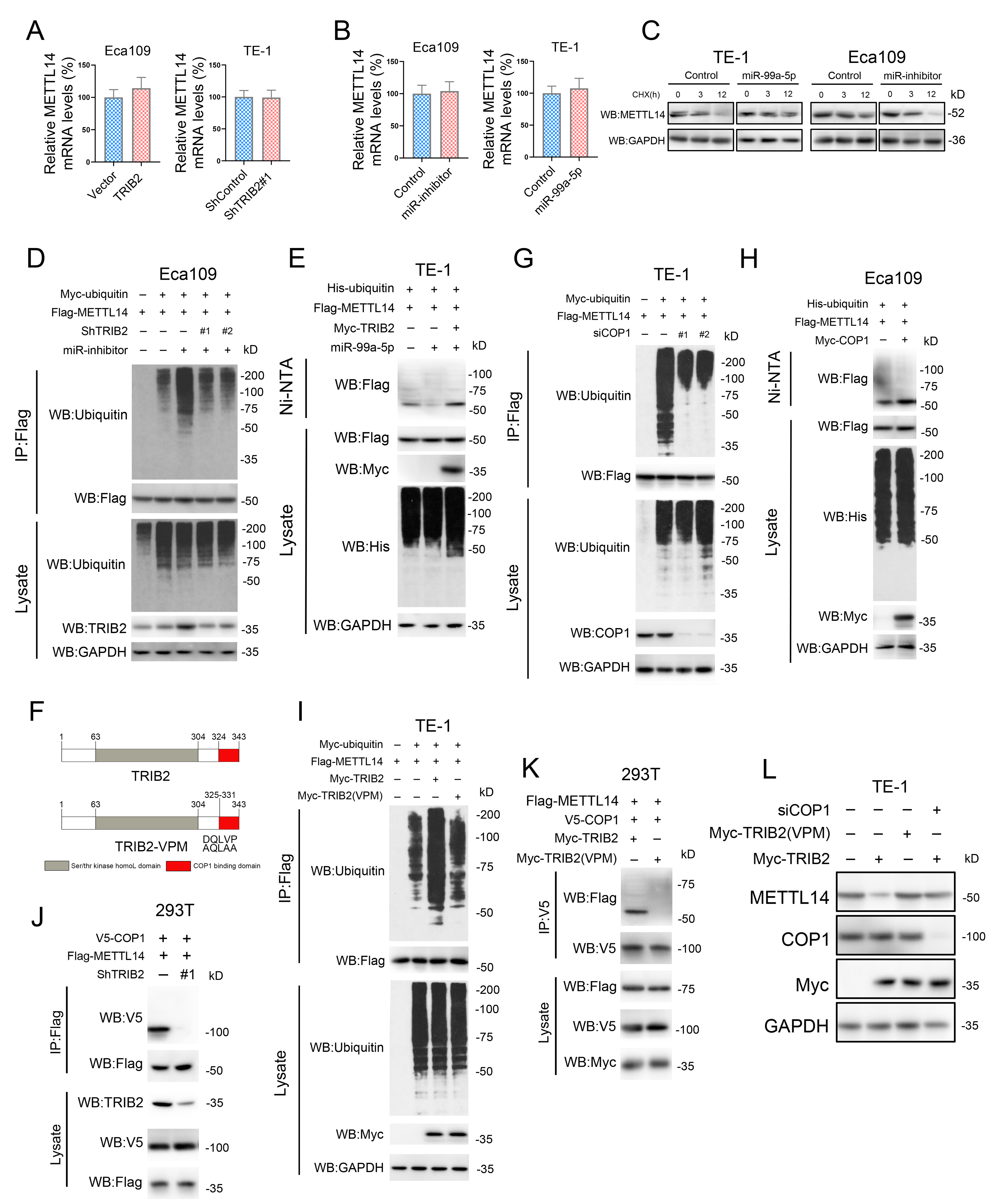

Supplement: Supplementary file 6 — SUPPORTING INFORMATION [file CTM2-11-e545-s011.tif]

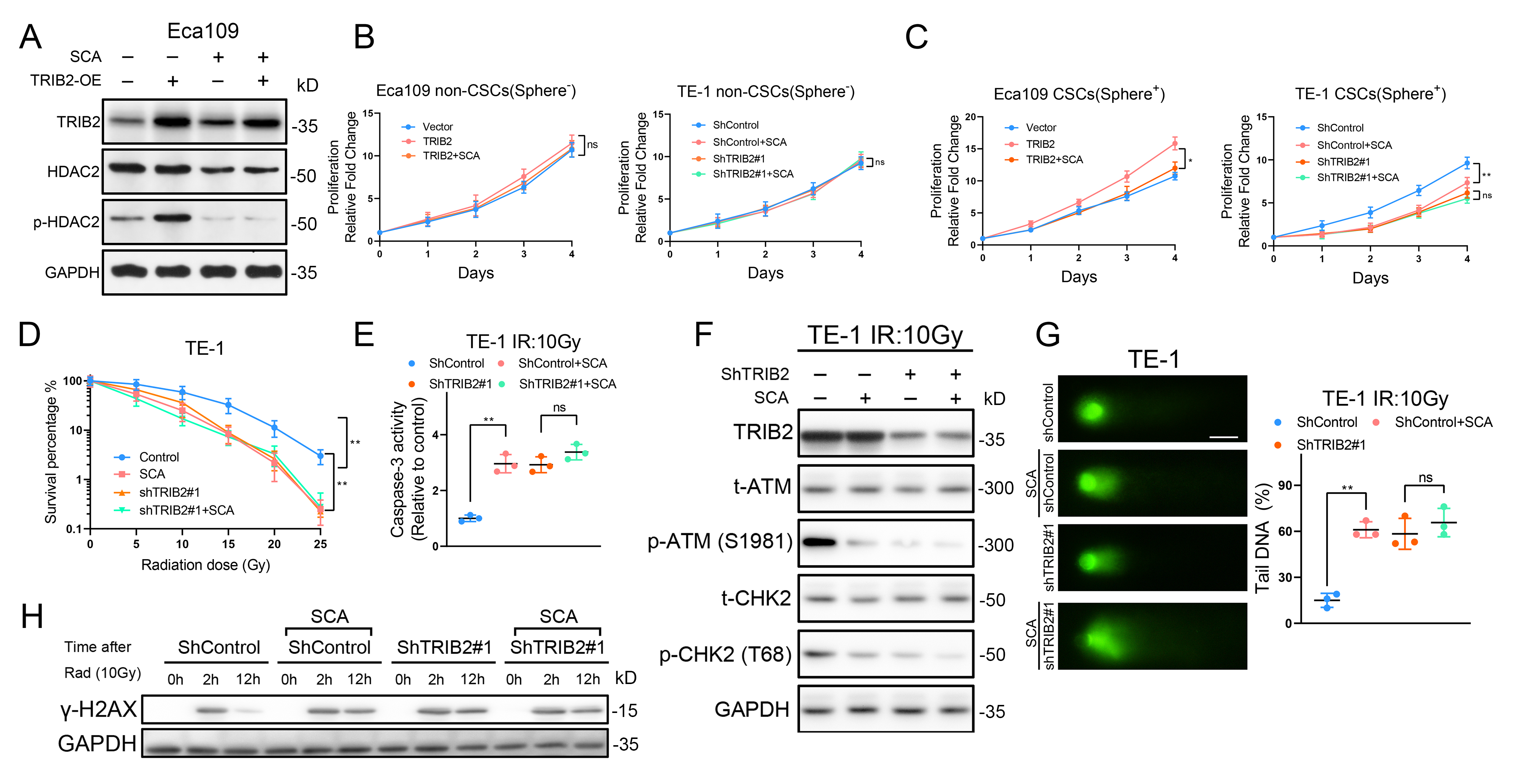

Supplement: Supplementary file 7 — SUPPORTING INFORMATION [file CTM2-11-e545-s009.tif]
