# Supplementary material for: A methyltransferase‐like 14/miR‐99a‐5p/tribble 2 positive feedback circuit promotes cancer stem cell persistence and radioresistance via histone deacetylase 2‐mediated epigenetic modulation in esophageal squamous cell carcinoma
Source: Clin Transl Med. 2021 Sep 15;11(9):e545. doi: 10.1002/ctm2.545 (PMC8441142; doi:10.1002/ctm2.545)
Supplement: Supplementary file 12 — SUPPORTING INFORMATION [file CTM2-11-e545-s005.docx]

**Supplementary Tables**

**Supplementary Table S5.** Clinicopathological features of ESCC patients in cohort I.

|  | **n = 26**  **n (%)** |
| --- | --- |
| **Age**  Median (range) | 64 (51-78) |
| **Sex**  Male  Female | 19 (73.1)  7 (26.9) |
| **Tumor location**  Upper  Middle  Lower | 8 (30.8)  8 (30.8)  10 (38.5) |
| **pT**  1  2  3  4 | 9 (34.6)  12 (46.2)  4 (15.4)  1 (3.8) |
| **pN**  0  1  2  3 | 11 (42.3)  8 (30.8)  6 (23.1)  1 (3.8) |
| **pM**  0  1 | 26 (100)  0 (0) |
| **TNM stage**  I  II  III  IV | 9 (34.6)  11 (42.3)  4 (15.4)  2 (7.7) |

**Supplementary Table S6.** Clinicopathological features of ESCC patients in cohort II.

|  | **n = 86**  **n (%)** |
| --- | --- |
| **Age**  Median (range) | 58 (46-75) |
| **Sex**  Male  Female | 51 (73.1)  35 (26.9) |
| **Tumor location**  Upper  Middle  Lower | 24 (30.8)  36 (30.8)  26 (38.5) |
| **pT**  1  2  3  4 | 27 (34.6)  25 (46.2)  26 (15.4)  6 (3.8) |
| **pN**  0  1  2  3 | 44 (42.3)  21 (30.8)  17 (23.1)  4 (3.8) |
| **pM**  0  1 | 81 (94.2)  5 (5.8) |
| **TNM stage**  I  II  III  IV | 30 (34.6)  22 (42.3)  19 (15.4)  15 (7.7) |

**Supplementary Table S7.** Clinicopathological features of ESCC patients in cohort III.

|  | **n = 78**  **n (%)** |
| --- | --- |
| **Age**  Median (range) | 60 (39-81) |
| **Sex**  Male  Female | 53 (67.9)  25 (23.1) |
| **Tumor location**  Upper  Middle  Lower | 18 (23.1)  34 (43.6)  26 (33.3) |
| **pT**  3  4 | 49 (62.8)  29 (37.2) |
| **pN**  0  > 0 | 28 (35.9)  50 (64.1) |
| **pM**  0  1 | 69 (88.5)  9 (11.5) |
| **TNM stage**  III  IV | 56 (71.8)  22 (28.2) |

**Supplementary Table S8.** Sequences of oligonucleotides used in the study.

| **Sequence name** | | **Sequence (5'-3')** |
| --- | --- | --- |
| **Real-time PCR primers:** | |  |
| NANOG sense | | CATGAGTGTGGATCCAGCTTG |
| NANOG antisense | | CCTGAATAAGCAGATCCATGG |
| OCT4 sense | | AGTGAGAGGCAACCTGGAGA |
| OCT4 antisense | | ACACTCGGACCACATCCTTC |
| BMI-1 sense | | TCGTTGTTCGATGCATTTCT |
| BMI-1 antisense | | CTTTCATTGTCTTTTCCGCC |
| ABCG2 sense | | TGGTGTTTCCTTGTGACACTG |
| ABCG2 antisense | | TGAGCCTTTGGTTAAGACCG |
| CD90 sense | | CGCTCTCCTGCTAACAGTCTT |
| CD90 antisense | | CAGGCTGAACTCGTACTGGA |
| CD271 sense | | ACGGCTACTACCAGGATGAG |
| CD271 antisense | | TGGCCTCGTCGGAATACGTG |
| TRIB2 sense | | CCGCCTACCCCACTTTTCAT |
| TRIB2 antisense | | TGTGGGACAACCTGCTTTGT |
| ALDH1 sense | | CTGTGTTCCAGGAGCCGAAT |
| ALDH1 antisense | | AGCATCCATAGTACGCCACG |
| SOX2 sense | | CAAGATGCACAACTCGGAGA |
| SOX2 antisense | | GCTTAGCCTCGTCGATGAAC |
| GAPDH sense | | GCACCGTCAAGGCTGAGAAC |
| GAPDH antisense | | TGGTGAAGACGCCAGTGGA |
| METTL14 sense | | AATGGCCGTTCTGTGCTCAT |
| METTL14 antisense | | AAGGACCCATCACAGGCAAG |
| p21 sense | | AGTCAGTTCCTTGTGGAGCC |
| p21 antisense | | AGGAGAACACGGGATGAGGA |
| pri-mir-99a sense | | CGTCTACCCTCATTCCCACG |
| pri-mir-99a antisense | | TGGGACACAAACTGCCCAAT |
| pre-miR-99a sense | | ACAGCCATCGTCCTTTCACTT |
| pre-miR-99a antisense | AGCCTTGGAAGACTCACAGG | |
| miR-101-3p sense | | TCGGCAGGTACAGTACTGTGATAA |
| miR-101-3p antisense | | CTCAACTGGTGTCGTGGA |
| miR-218-5p sense | | TCGGCAGGTTGTGCTTGATCTAACC |
| miR-218-5p antisense | | CTCAACTGGTGTCGTGGA |
| miR-140-3p sense | | TCGGCAGGTACCACAGGGTAGAA |
| miR-140-3p antisense | | CTCAACTGGTGTCGTGGA |
| miR-29c-3p sense | | GCCGAGTAGCACCATTTGAAATCG |
| miR-29c-3p antisense | | CTCAACTGGTGTCGTGGA |
| miR-99a-5p sense | | TCGGCAGGAACCCGTAGATCCGATC |
| miR-99a-5p antisense | | CTCAACTGGTGTCGTGGA |
| 5s rRNA sense | | TACGGCCATACCACCCTGAAC |
| 5s rRNA antisense | | CGGTCTCCCATCCAAGTACTAACC |
| promoter region of p21 sense | | TCCTCTGAAAGCTGACTGCC |
| promoter region of p21 antisense | | CAGCATGGGGTAGGGGAATG |
| **shRNAs:** | |  |
| ShTRIB2#1 | | CAACCACCTAGCTGGTAATTA |
| ShTRIB2#2 | | GCATCGCACTGTTAGCATTTA |
| ShMETTL14#1 | | GCTTACAAATAGCAACTACAA |
| ShMETTL14#2 | | CCATGTACTTACAAGCCGATA |
| **siRNAs:** | |  |
| siCOP1 | | ACTCTCAGCAGGTCCGATACC |
| siAkt | | GCACCTTCATTGGCTACAAGG |
| siS6K1 | | TATTTGCCATGAAGGTGCTTA |
| siMETTL3 | | GCAAGTATGTTCACTATGAAA |
| siFTO | | TCACGAATTGCCCGAACATTA |
| siWTAP | | GCACGGGATGAGTTAATTCTA |
| siALKBH5 | | GCTGTCTTCAAGTGGAAGTTT |
| siDGCR8 | | CGAGCTGAAGAAGTCTGGTTT |

**Supplementary Figures**

**Supplementary Figure Legends**

**Figure S1. Identification of the stemness of enriched spheres of ESCC cells.**

**A-C.** The relative expression of various CSC markers (ALDH1, SOX2, OCT4, BMI-1, CD90, CD271, ABCG-2 and NANOG) in enriched spheres and non-spheres of Eca109 (**A**), TE-1 (**B**) and Kyse150 (**C**) measured by qRT-PCR. **D.** Correlation between miR-99a-5p and CD90 mRNA levels in patients in Cohort I, as detected by qPCR. The data are presented as the mean ± SD. *P < 0.05, **P < 0.01, ***P < 0.001. P values were determined using unpaired Student’s t test (**A**, **B** and **C**). The correlation was determined by the Pearson correlation test (**D**).

**Figure S2. Effects of miR-99a-5p on the CSC properties and radioresistance of ESCC cells.**

**A.** Representative images (left) and statistical quantification (right) of the flow cytometric analysis used to determine the proportion of CD90^+^ cells among the indicated ESCC cells. **B.** Representative images (left) and statistical quantification (right) of the flow cytometric analysis used to determine the proportion of CD271^+^ cells among the indicated ESCC cells. **C.** The proportion of CD90^+^ subpopulations in ESCC cells before, 0 days after and 5 days after cellular sorting was determined by a flow cytometer. **D-E.** Cell viability of the CD90^-^ (**D**) and CD271^-^ (**E**) enriched cell populations of the indicated ESCC cells. **F.** Statistical analysis of the ISH results in Figure 2F. **G.** Quantification of the blot intensity relative to GADPH expression of the western blot in Figure 2K. **H.** Representative images and statistical quantification of the comet assay results in non-CSCs of ESCC cells with miR-99a-5p overexpression or inhibition 24 hours after IR at a dose of 10 Gy. Scale bars: 10 μm. **I.** Colony formation assays of non-CSCs of ESCC cells with miR-99a-5p overexpression or inhibition after IR at a dose of 10 Gy. **J.** Relative caspase-3 activity in non-CSCs of ESCC cells with miR-99a-5p overexpression or inhibition 24 hours after IR at a dose of 10 Gy. The data are presented as the mean ± SD. *P < 0.05, **P < 0.01, ***P < 0.001. P values were determined by unpaired Student’s t tests (**A, B, D, E, H, I** and **J**) and Mann Whitney tests (**F**).

**Figure S3. m^6^A regulates miR-99a-5p expression in ESCC cells.**

**A.** The transcription start site (TSS) of pri-mir-99a was determined by the 5′ RACE analysis. The framed sequence represents primers used for 5′ RACE analysis. The sequence in red shows the sequence of miR-99a-5p and miR-99a-3p. GSP, gene specific-primer. **B.** A series of fragments upstream pri-mir-99a TSS with progressive 5′ deletions were cloned in front of firefly luciferase reporter gene. HEK293T cells were cotransfected with above constructed plasmids along with control qRL-TK plasmid. Luciferase assays showed that the transcriptional activity was markedly increased after transfection with the promoter reporters P-1500, P-2500 and P-5000 compared with the negative control plasmid. The luciferase activity of these three reporters had no significant differences, indicating the promoter region of pri-mir-99a is located between positions −1500 to +50. **C.** The putative promoter region of pri-mir-99a coincides with the occurrence of H3K4Me3 and H3K27Ac, two histone modification biomarkers closely associated with promoter and regulatory element regions, illustrated by the UCSC Genome Browser. **D.** The promoter activity of pri-mir-99a in spheres and nonspheres of ESCC cells was measured by luciferase assays. **E.** ESCC cells were treated with specific inhibitors of HDAC1 (CI994, 10 μM), HDAC2 (SCA, 20 μM), HDAC3 (GFRP966, 10 μM), HDAC4 (tasquinimod, 10 μM), HDAC6 (CAY1215, 4 μM), and HDAC8 (PCI34051, 5 μM) and a broad-spectrum HDAC inhibitor (SAHA2, μM), and miR-99a-5p expression was assessed by qRT-PCR. **F.** The sphere and nonsphere subpopulations of Eca109 cells were treated with Act-D (5 μg/ml) for the indicated time periods, and the levels of pri-mir-99a and mature miR-99a-5p were measured by qRT-PCR. **G.** SRAMP prediction indicated there are two m^6^A modification sites with high confidence along full length pri-mir-99a. **H.** The efficiency of silencing of METTL14 by shRNA in Eca109 cells as detected by western blotting. **I.** The efficiency of silencing of METTL3 by siRNA in Eca109 cells, as detected by western blotting. **J.** miR-99a-5p expression was measured by RT-PCR after transfection of Eca109 cells with the indicated siRNAs. **K.** Levels of METTL14 in ESCC cells transfected with METTL14-expressing lentivirus in ESCC cells, as detected by western blotting. **L.** The promoter activity of pri-mir-99a in ESCC after METTL14 knockdown or overexpression was measured by luciferase assays. *P < 0.05, **P < 0.01, ***P < 0.001. P values were determined by one-way ANOVA with Tukey’s post hoc test (**E** and **L**) or unpaired Student’s t test (**D, F, J** and **L**).

**Figure S4. METTL14 promotes pri-mir-99a preprocessing in a GDCR8-dependent manner.**

**A.** The expression of precursor mir-99a in the cytoplasm and nucleus was measured by RT-PCR. **B.** ESCC cells were treated with Act-D (5 μg/ml) for the indicated time periods, and miR-99a-5p expression was measured by RT-PCR. **C.** Levels of DGCR8 in ESCC cells transfected with a DGCR8-expressing plasmid and efficiency of silencing of DGCR8 by siRNA in ESCC cells, as detected by western blotting. **D.** Relative pri-mir-99a RNA level in the input sample of Figure 3N determined by qPCR. **E.** The relative expression of pri-mir-99a in the indicated ESCC cells transfected with the indicated constructs was determined by qPCR. The data are presented as the mean ± SD. *P < 0.05, **P < 0.01, ***P < 0.001. P values were determined by one-way ANOVA with Tukey’s post hoc test (**B** and **E**) or unpaired Student’s t test (**A** and **B**).

**Figure S5. The suppressive effect of miR-99a-5p on CSC properties and radioresistance is through inhibiting TRIB2.**

**A.** Western blotting analysis of CSC markers in the indicated ESCC cells. **B.** Representative images (left) and statistical quantification (right) of sphere formation by the indicated ESCC cells. Scale bars: 100 μm. **C-D.** Representative images and statistical quantification of the flow cytometric analyses to determine the ALDH activity and the relative proportions of CD90^+^ and CD271^+^ subpopulations in the indicated ESCC cells. **E.** Clonogenic assay of TE-1 cells transfected with indicated constructs after an IR dose of 10 Gy. The survival fraction was calculated as: (number of colonies formed/number of cells plated) _irradiated_/ (number of colonies formed/number of cells plated) _control_. **F.** Relative caspase-3 activity of the indicated ESCC cells 24 hours after an IR dose of 10 Gy. **G.** Western blotting analysis of the phosphorylated and total amounts of the checkpoint markers ATM and CHK in the indicated ESCC cells 4 hours after an IR dose of 10 Gy. **H.** Representative images (left) and statistical quantification (right) of the comet assay results in the indicated ESCC cells 6 hours after an IR dose of 10 Gy. Scale bars: 10 μm. The data are presented as the mean ± SD. *P < 0.05, **P < 0.01, ***P < 0.001. P values were determined by one-way ANOVA with Tukey’s post hoc test (**B, D, E, F** and **H**).

**Figure S6. TRIB2 functions as a substrate adaptor of COP1 upon the ubiquitination of METTL14.**

**A-B.** METTL14 mRNA expression levels in the indicated ESCC cells were measured by RT-PCR. **C**. Western blotting analysis of the stability of METTL14 in TE-1 cells transfected with miR-99a-5p mimics and in Eca109 cells transfected with an miR-99a-5p inhibitor. **D**. IP analysis of METTL14 ubiquitination in Eca109 cells transfected with the indicated constructs, miR-99a-5p inhibitor and/or TRIB2 shRNA. **E.** Ni-NTA pull-down analysis of METTL14 ubiquitination in TE-1 cells transfected with the indicated constructs and/or with miR-99a-5p mimics. **F**. Schematic showing the TRIB2 point mutants. The amino acid sequences show the original sequence on top and the mutated sequence on the bottom. **G.** IP analysis of METTL14 ubiquitination in TE-1 cells transfected with the indicated constructs and/or with siCOP1. **H.** Ni-NTA pull-down analysis of METTL14 ubiquitination in Eca109 cells transfected with the indicated constructs. **I.** IP analysis of METTL14 ubiquitination in TE-1 cells transfected with the indicated constructs. **J.** IP analysis of the interaction between COP1 and METTL14 in 293T cells transfected with the COP1 construct and/or with TRIB2 shRNA. **K.** IP analysis of the interaction between COP1 and METTL14 in 293T cells transfected with TRIB2 or with its mutant (VPM) construct. **L**. Western blotting analysis of the expression of METTL14 in TE-1 cells transfected with the indicated constructs and/or with siCOP1. The data are presented as the mean ± SD. *P < 0.05, **P < 0.01, ***P < 0.001. P values were determined by unpaired Student’s t test.

**Figure S7. HDAC2 is essential for the TRIB2-mediated CSC properties and radioresistance of ESCC cells.**

**A.** The levels of total and phosphorylated HDAC2 in Eca109 cells with or without SCA (2 μM) treatment were measured by western blotting. **B-C.** Cell viability of the Sphere^-^- (**B**) and Sphere^+^- (**C**) enriched cell populations present in the indicated ESCC cells. **D.** Clonogenic assay of the indicated ESCC cells with or without an HDAC2 inhibitor (SCA, 2 μM) at an IR dose of 10 Gy. **E.** Relative caspase-3 activity of the indicated ESCC cells 24 hours after an IR dose of 10 Gy. **F**. Western blotting analysis of the phosphorylated and total amounts of the checkpoint markers ATM and CHK in the indicated ESCC cells 4 hours after an IR dose of 10 Gy. **G.** Representative images (left) and statistical quantification (right) of the comet assay results for the indicated ESCC cells 6 hours after an IR dose of 10 Gy. Scale bars: 10 μm. **H.** The indicated ESCC cells were cultured under normal conditions for 2 or 12 hours after receiving an IR dose of 10 Gy, and the expression of γ-H2AX in the cells was then measured by western blotting. The data are presented as the mean ± SD. *P < 0.05, **P < 0.01, ***P < 0.001. P values were determined by one-way ANOVA with Tukey’s post hoc test.
